# Supplementary material for: Structure of the Ribosomal Oxygenase OGFOD1 Provides Insights into the Regio- and Stereoselectivity of Prolyl Hydroxylases
Source: Structure. 2015 Apr 7;23(4):639–52. doi: 10.1016/j.str.2015.01.014 (PMC4396695; doi:10.1016/j.str.2015.01.014)
Supplement: Document S1. Figures S1 and S2 and Table S1 [file mmc1.pdf]

Structure, Volume *23*

Supplemental Information

Structure of the Ribosomal Oxygenase OGFOD1 Provides Insights into the Regio- and Stereoselectivity of Prolyl Hydroxylases

Shoichiro Horita, John S. Scotti, Cyrille Thinnes, Yousef S. Mottaghi-Taromsari, Armin Thalhammer, Wei Ge, WeiShen Aik, Christoph Loenarz, Christopher J. Schofield, and Michael A. McDonough

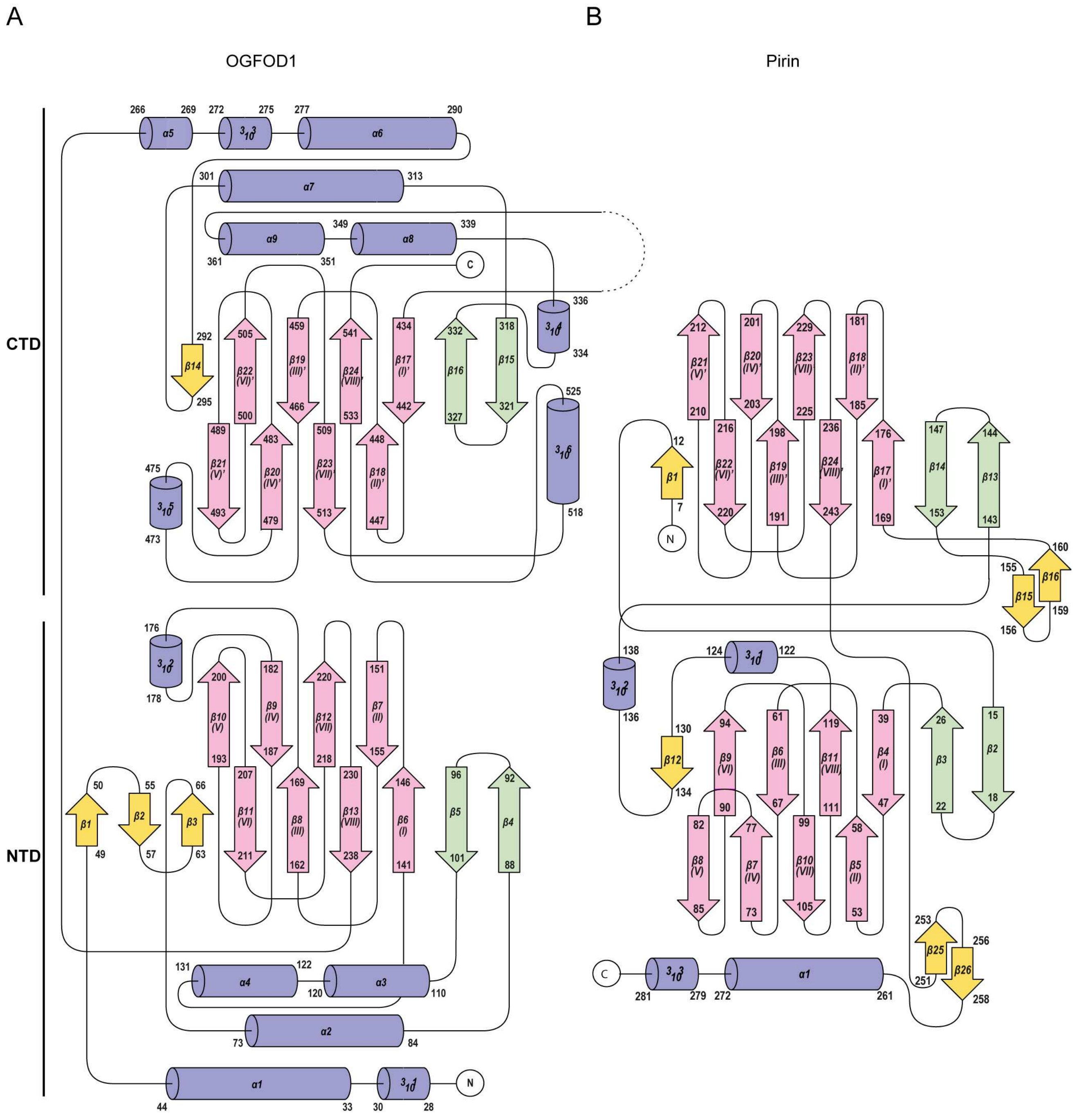

Figure S1, related to Figure 2. Topological fold diagrams of (A) OGFOD1 (PDB ID: 4NHX) and (B) Pirin (Pang et al., 2004) (PDB ID: 1J1L), α-helix and 3_{10}-helix (blue), β-strand (pink/green/yellow). The eight β-strands forming the core DSBH fold (pink), the β-strands forming the β4-β5 hairpin (green), all other strands (yellow) are shown. The eight DSBH β-strands of OGFOD1 are additionally labelled with Roman numerals as in Clifton *et al.* (Clifton et al., 2006). The long disordered 'acidic' loop is represented by a dashed line.

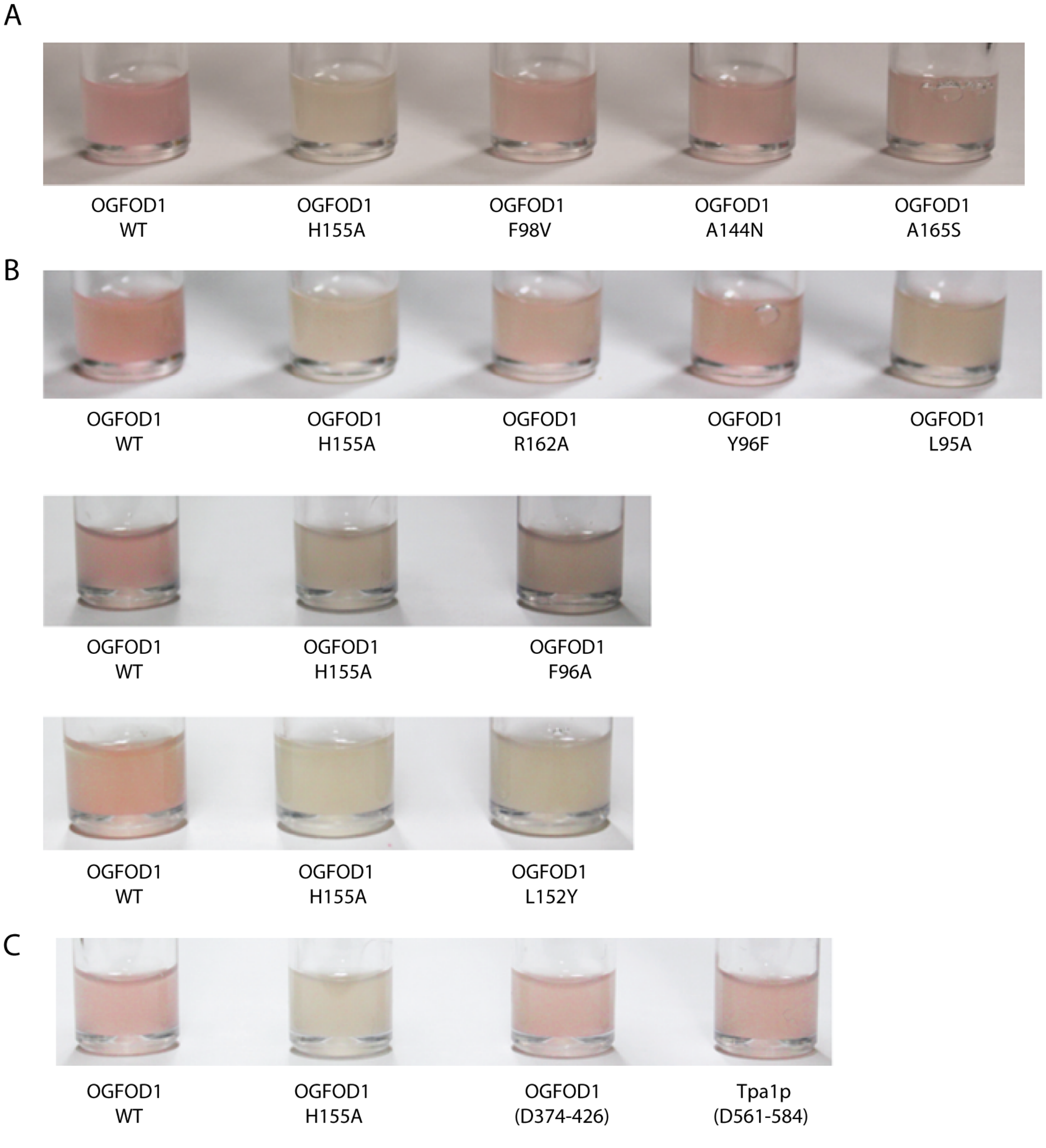

Figure S2, related to Figure 7. Yeast cell assays of OGFOD1 and Tpa1p variants for assessing yeast uS12 *trans*-3-hydroxylation. Di-hydroxylation of Pro64 was observed for Tpa1p activity but not OGFOD1 (Loenarz et al., 2014). We analysed the structures for active site differences between OGFOD1 and Tpa1p in pursuit of identifying residues responsible for mono- versus di-hydroxylation. Substitution of OGFOD1 residues to the corresponding Tpa1p residues (F98V, A144N, and A165S) (within 11 Å of the active site metal in the substrate binding groove) (Figure 7I) did not appear to affect *trans*-P3H activity in the cellular assays, suggesting these changes are tolerated (A) and that lack of di-hydroxylation activity by OGFOD1 may be influenced by factors other than/additional to immediate active site differences.

Table S1, related to Table 1. Details of crystallization conditions.

	OGFOD1:NOG[a]	OGFOD1:2,4-PDCA[a]	Tpa1p:NOG[a]	Tpa1p:2,4-PDCA[a]	Tpa1p:IOX3[a]
**Protein solution**	~10 mg/mL OGFOD1, 0.7 mM $MnCl_2$, 1.0 mM NOG, 1.0 mM C036 peptide	~10 mg/mL OGFOD1, 2.0 mM $MnCl_2$, 2.0 mM 2,4-PDCA	~9.5 mg/mL TPA1, 0.8 mM $MnCl_2$, 1.1 mM NOG	~6 mg/mL TPA1, 0.8 mM $MnCl_2$, 1.1 mM 2,4-PDCA	~6 mg/mL TPA1, 0.8 mM $MnCl_2$, 1.1 mM IOX3
**Reservoir solution**	0.1 M MIB buffer pH 6.5, 0.1 M glycine, 25% PEG 1500	0.1 M HEPES pH 7.0, 0.2 M $MgCl_2$, 20% PEG 6000	0.1 M succinic acid pH 7.0, 12% PEG 3350	0.1 M SPG buffer pH 8.0, 25% PEG 1500	0.2 M trisodium citrate, 20% PEG 3350

Abbreviations: PEG; polyethylene glycol, **SPG** buffer; succinic acid, phosphate, glycine, **MIB** buffer; malonic acid, imidazole, boric acid. C036 peptide; LEKLGIESKQPNSAIRKAVR($_D$-Cys)($_D$-Cys, $_D$-cysteine residue).
[a] All crystals were soaked in cryoprotectant containing reservoir solution diluted with 25% glycerol.

REFERENCES

Clifton, I.J., McDonough, M.A., Ehrismann, D., Kershaw, N.J., Granatino, N., Schofield, C.J. (2006). Structural studies on 2-oxoglutarate oxygenases and related double-stranded β-helix fold proteins. J. Inorg. Biochem. *100*, 644-669.

Loenarz, C., Sekirnik, R., Thalhammer, A., Ge, W., Spivakovsky, E., Mackeen, M.M., McDonough, M.A., Cockman, M.E., Kessler, B.M., Ratcliffe, P.J., et al. (2014). Hydroxylation of the eukaryotic ribosomal decoding centre affects translational accuracy. Proc. Natl. Acad. Sci. USA *111*, 4019-4024.

Pang, H., Bartlam, M., Zeng, Q., Miyatake, H., Hisano, T., Miki, K., Wong, L.L. Gao, G.F., Rao, Z. (2004). Crystal structure of human Pirin: an iron-binding nuclear protein and transcription cofactor. J. Biol. Chem. *279*, 1491-1498.
