# Supplementary material for: Structure of the Ribosomal Oxygenase OGFOD1 Provides Insights into the Regio- and Stereoselectivity of Prolyl Hydroxylases
Source: Structure. 2015 Apr 7;23(4):639–52. doi: 10.1016/j.str.2015.01.014 (PMC4396695; doi:10.1016/j.str.2015.01.014)
Supplement: Document S2. Article plus Supplemental Information [file mmc7.pdf]

Structure

Article

Structure of the Ribosomal Oxygenase OGFOD1 Provides Insights into the Regio- and Stereoselectivity of Prolyl Hydroxylases

Graphical Abstract

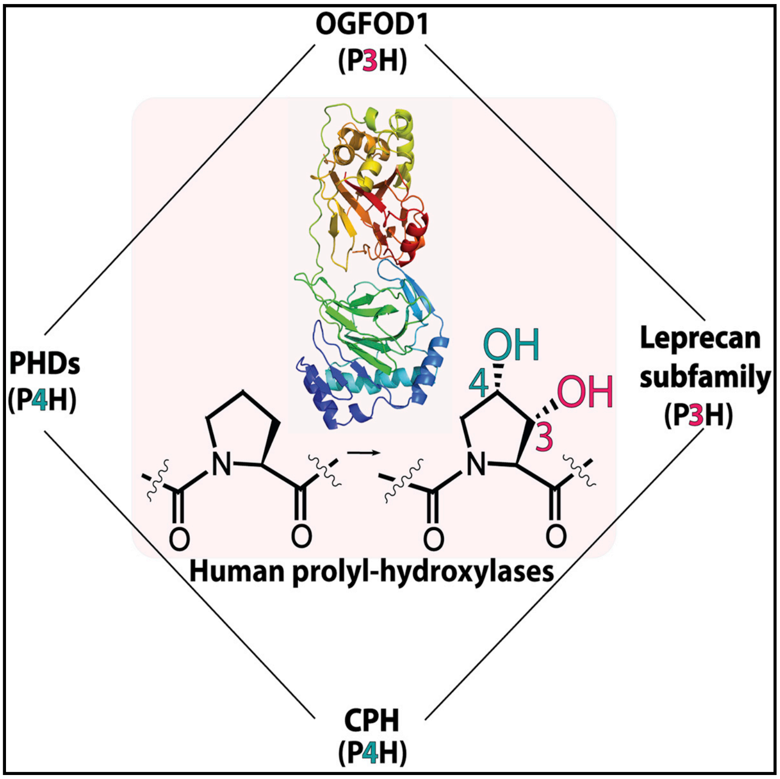

Authors

Shoichiro Horita, John S. Scotti, ...,
Christopher J. Schofield,
Michael A. McDonough

Correspondence

christopher.schofield@chem.ox.ac.uk
(C.J.S.),
michael.mcdonough@chem.ox.ac.uk
(M.A.M.)

In Brief

Horita et al. present structures of the first prolyl 3-hydroxylase from humans, OGFOD1, in complex with small-molecule inhibitors. The results shed light on the catalytic mechanisms and evolution of the OGFOD1 subfamily and the related “oxygen-sensing” hypoxia-inducible factor *trans*-P4Hs (PHDs).

Highlights

- Crystal structures of human OGFOD1 in complex with inhibitors are presented
- The OGFOD1 and PHDs likely share a common ancestor
- The OGFOD1 active site is comparable with hypoxia-inducible factor prolyl hydroxylase
- Differences between OGFOD1 and PHD2 can be exploited for inhibitor design

Accession Numbers

4NHX
4NHY
4NHL
4NHK
4NHM

Horita et al., 2015, Structure *23*, 639–652
April 7, 2015 ©2015 The Authors
http://dx.doi.org/10.1016/j.str.2015.01.014

Structure
Article

Structure of the Ribosomal Oxygenase OGFOD1 Provides Insights into the Regio- and Stereoselectivity of Prolyl Hydroxylases

Shoichiro Horita,[1,2,3] John S. Scotti,[1,3] Cyrille Thinnes,[1] Yousef S. Mottaghi-Taromsari,[1] Armin Thalhammer,[1] Wei Ge,[1] WeiShen Aik,[1] Christoph Loenarz,[1] Christopher J. Schofield,[1,*] and Michael A. McDonough[1,*]
[1]Chemistry Research Laboratory, Department of Chemistry, University of Oxford, 12 Mansfield Road, Oxford OX1 3TA, UK
[2]Department of Physiology, Anatomy and Genetics, University of Oxford, Parks Road, Oxford OX1 3PT, UK
[3]Co-first author
*Correspondence: christopher.schofield@chem.ox.ac.uk (C.J.S.), michael.mcdonough@chem.ox.ac.uk (M.A.M.)
http://dx.doi.org/10.1016/j.str.2015.01.014
This is an open access article under the CC BY license (http://creativecommons.org/licenses/by/4.0/).

SUMMARY

Post-translational ribosomal protein hydroxylation is catalyzed by 2-oxoglutarate (2OG) and ferrous iron dependent oxygenases, and occurs in prokaryotes and eukaryotes. OGFOD1 catalyzes *trans*-3 prolyl hydroxylation at Pro62 of the small ribosomal subunit protein uS12 (RPS23) and is conserved from yeasts to humans. We describe crystal structures of the human uS12 prolyl 3-hydroxylase (OGFOD1) and its homolog from *Saccharomyces cerevisiae* (Tpa1p): OGFOD1 in complex with the broad-spectrum 2OG oxygenase inhibitors; *N*-oxalylglycine (NOG) and pyridine-2,4-dicarboxylate (2,4-PDCA) to 2.1 and 2.6 Å resolution, respectively; and Tpa1p in complex with NOG, 2,4-PDCA, and 1-chloro-4-hydroxyisoquinoline-3-carbonylglycine (a more selective prolyl hydroxylase inhibitor) to 2.8, 1.9, and 1.9 Å resolution, respectively. Comparison of uS12 hydroxylase structures with those of other prolyl hydroxylases, including the human hypoxia-inducible factor (HIF) prolyl hydroxylases (PHDs), reveals differences between the prolyl 3- and prolyl 4-hydroxylase active sites, which can be exploited for developing selective inhibitors of the different subfamilies.

INTRODUCTION

The discovery that collagen biosynthesis involves oxygenase catalyzed hydroxylation of prolyl residues was important because it expanded the field of post-translational modifications. Subsequently, C-3 and C-4 prolyl hydroxylations were identified in collagen-like domains of multiple other proteins and non-protein natural products (Hausinger, 2004; Myllyharju and Kivirikko, 2004; Gorres and Raines, 2010). More recently, prolyl 4-hydroxylation of hypoxia-inducible factor (HIF) α subunits as catalyzed by the HIF prolyl hydroxylases (PHDs or EGLNs) has been shown to play a central role in enabling the hypoxic response in humans and other animals (Kaelin and Ratcliffe, 2008; Schofield and Ratcliffe, 2004). All identified C-3 and C-4 prolyl hydroxylases (P3H and P4H, respectively) are part of the 2-oxoglutarate (2OG) and ferrous iron dependent oxygenase superfamily (Hausinger, 2004; Loenarz and Schofield, 2008, 2011). 2OG oxygenases catalyze hydroxylation (and *N*-demethylation via hydroxylation) reactions of a diverse set of substrates, and in eukaryotes have roles in metabolism, transcriptional regulation, epigenetics, and nucleic acid modification and repair (Hausinger, 2004; Schofield and Ratcliffe, 2004; Klose et al., 2006; Falnes et al., 2007; Loenarz and Schofield, 2008, 2011; Jia et al., 2013).

The range of functions identified for 2OG oxygenases has recently expanded to include the post-translational modification of ribosomal and ribosome-associated proteins as catalyzed by ribosomal oxygenases (ROXs) (Ge et al., 2012; Feng et al., 2013; Katz et al., 2014; Loenarz et al., 2014; Singleton et al., 2014; Figure 1). OGFOD1 in *Homo sapiens*, Sud1 in *Drosophila melanogaster*, and Tpa1p in *Saccharomyces cerevisiae* were found to catalyze prolyl 3-hydroxylation of the highly conserved Pro62 and Pro64 of uS12 (RPS23) in humans and yeast, respectively (Ban et al., 2014; Katz et al., 2014; Loenarz et al., 2014; Singleton et al., 2014). Although the biological roles for uS12 hydroxylation are still emerging, it has been reported that in yeast it can regulate translation in a sequence context dependent manner and that it is involved in stress responses (Saito et al., 2010; Katz et al., 2014; Loenarz et al., 2014; Singleton et al., 2014). Ofd1, a homolog of OGFOD1/Tpa1p from *Schizosaccharomyces pombe*, binds to the helical repeat protein Nro1 in an O_2-dependent manner (Rispal et al., 2011; Yeh et al., 2011), thus inhibiting Ofd1 binding to Sre1N, a homolog of the sterol regulatory element binding protein (Lee et al., 2009). In humans, a distinct ROX subfamily, more closely related to the JmjC domain subfamily, comprising MYC-induced nuclear antigen 53 kDa (MINA53) and nucleolar protein 66 kDa (NO66) catalyzes histidyl hydroxylation of human ribosomal proteins uL15 (L27A) and uL2 (L8), respectively. A prokaryotic homolog of MINA53/NO66, YcfD, catalyzes arginyl hydroxylation of ribosomal protein uL16 (L16) (Ge et al., 2012; Figure 1). These findings have led to the proposal that 2OG oxygenases are widespread regulators of ribosomal processivity, translation rate, and translational accuracy (Keeling et al., 2006; Wehner et al., 2010; Katz et al., 2014; Loenarz et al., 2014; Singleton et al., 2014).

The first human prolyl hydroxylase (PH) crystal structures to be reported were of the HIF PH (PHD2) (McDonough et al.,

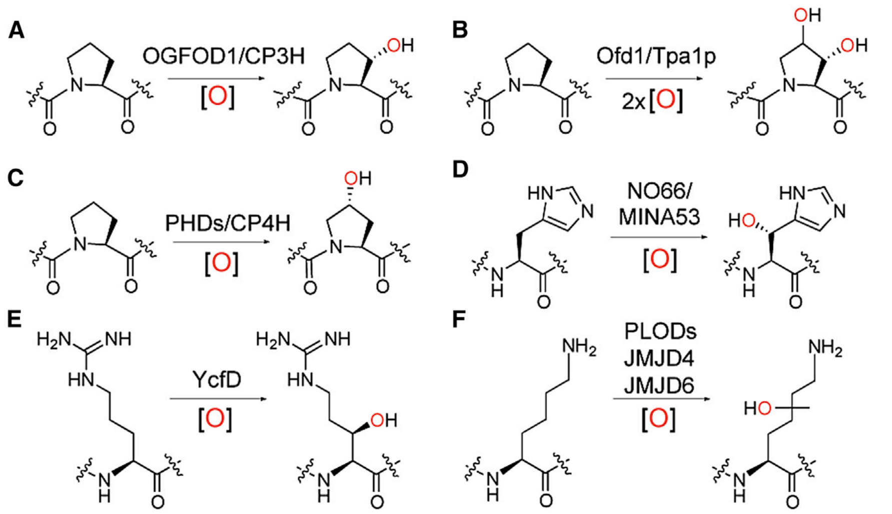

Figure 1. Post-Translational Hydroxylations Catalyzed by Ribosomal Protein Prolyl- and Related 2-Oxoglutarate Oxygenases

(A and B) OGFOD1 catalyzes *trans* C-3 prolyl hydroxylation (A), whereas yeast Tpa1p and Ofd1 catalyze *trans* C-3 and/or C-4 hydroxylations (B). The Leprecan subfamily of animal collagen PHs (CP3H) also catalyze C-3 prolyl hydroxylation (Vranka et al., 2004).

(C) The HIF PHs (PHDs) and collagen P4Hs (CP4Hs) catalyze *trans* C-4 prolyl hydroxylation (Gorres and Raines, 2010).

(D) MYC-induced nuclear antigen 53 (MINA53) and nucleolar protein 66 (NO66) are human ribosomal protein hydroxylases catalyzing C-3 histidyl hydroxylation.

(E) YcfD is a bacterial ribosomal hydroxylase that catalyzes C-3 arginyl hydroxylation.

(F) Lysyl hydroxylases with different regio- and stereoselectivities have been identified: pro-collagen lysyl hydroxylases (PLODs) (Myllyharju and Kivirikko, 2004), a eukaryotic release factor 1 (eRF1) hydroxylase (JMJD4) (Feng et al., 2013), and a splicing regulatory protein (U2AF) hydroxylase (JMJD6) (Webby et al., 2009).

All hydroxylations are coupled to the oxidation of 2OG to give succinate and CO_2.

2006). These studies revealed that the PHs contain a "distorted" double-stranded β helix (DSBH) fold characteristic of 2OG oxygenases, and possess a mobile β2-β3 "finger" loop and C-terminal helix that are important for substrate recognition. Structures of other PHs, including those acting on collagen-like proteins (Koski et al., 2007) and a recently identified bacterial *trans*-4 PH (PPHD) (Scotti et al., 2014), have led to the proposal that PHs comprise a distinctive subfamily of 2OG oxygenases that share a common ancestor (Eriksson et al., 1999; Clifton et al., 2001). Prior to its assignment as a uS12 P3H, crystal structures of Tpa1p revealed that it contains tandem DSBH domains, only one of which, the N-terminal domain, was predicted to harbor the catalytic machinery (Kim et al., 2009; Henri et al., 2010).

We report crystal structures of human OGFOD1 and its yeast homolog Tpa1p in complex with inhibitors. Combined with structurally informed activity analyses on active site variants in cells, the results provide a basis for a detailed molecular understanding of the regio- and stereoselectivity for different subfamilies of the PHs, further inform on their evolution, and will enable the design of selective PH inhibitors.

RESULTS

Following optimization of hits from high-throughput crystallization trials, diffraction quality crystals for full-length *H. sapiens* OGFOD1 (542 amino acids [aa], 63 kDa) and *S. cerevisiae* Tpa1p (644 aa, 74 kDa) in complex with Mn(II) and inhibitors were obtained (catalytically inactive Mn(II) was used as an Fe(II) surrogate) (Table S1). We determined structures for OGFOD1 and Tpa1p in complex with the broad-spectrum 2OG oxygenase inhibitors *N*-oxalylglycine (NOG) and pyridine-2,4-dicarboxylate (2,4-PDCA) (Rose et al., 2011). In addition, a structure of Tpa1p in complex with 1-chloro-4-hydroxyisoquinoline-3-carbonylglycine (IOX3), a 2OG competitive inhibitor closely related to compounds in clinical trials as a PHD inhibitor, was determined (Chowdhury et al., 2013).

OGFOD1 crystallized in both trigonal (OGFOD1:Mn(II):NOG) and orthorhombic (OGFOD1:Mn(II):2,4-PDCA) crystal systems with either one or four molecules per asymmetric unit (ASU), respectively. Tpa1p crystals were easier to obtain than OGFOD1 and were more amenable to crystallization with several inhibitors, including IOX3. Tpa1p crystals were monoclinic and contained one molecule in the ASU as for the reported Tpa1p structures (Kim et al., 2009; Henri et al., 2010). Structures were determined by molecular replacement using Tpa1p as a search model followed by iterative cycles of model fitting and refinement (Table 1).

We investigated the importance of selected residues of OGFOD1 and Tpa1p, using a yeast cell assay that qualitatively assesses the functional consequence of variants of OGFOD1 or Tpa1p catalyzed yeast uS12 prolyl hydroxylation (Loenarz et al., 2014). In wild-type *S. cerevisiae*, or *S. cerevisiae* in which the gene encoding for *TPA1* is replaced by *OGFOD1*, a decrease in uS12 hydroxylation activity leads to decreased synthesis of a red pigment due to the inhibition of nonsense codon read-through of an adenine biosynthesis gene (Namy et al., 2003).

Overall Architecture of OGFOD1

The structure of OGFOD1 consists of nine α helices, six 3_{10} helices, and 24 β strands that fold into two distinct DSBH domains (Figures 2 and S1A). The "catalytic" metal binding N-terminal domain (NTD, 24–238) and the C-terminal domain (CTD, 270–542) are connected by a linker region (239–269) and pack tightly against each other via their "minor" β sheets (as defined below).

The NTD of OGFOD1 contains six helices ($3_{10}1$, $3_{10}2$, α1–α4) and 13 β strands (β1–β13) (Figures 2 and S1A). The DSBH core of the NTD comprises 8 β strands (I–VIII), which form two β sheets (major and minor) that fold to form a β sandwich within which the 2OG binding pocket and metal binding site are located. Four antiparallel β strands (β7(II)-β12(VII)-β9(IV)-β10(V)) form the minor β sheet. Nine antiparallel β strands (β1-β2-β3-β11(VI)-β8(III)-β13(VIII)-β6(I)-β5-β4) form the major β sheet. β Strands β4 and β5 form a "hairpin" (β4-β5 hairpin) that outlines

Table 1. Crystallographic Data Collection and Refinement Statistics

Protein	OGFOD1 Mn^{2+} NOG	OGFOD1 Mn^{2+} 2,4-PDCA	Tpa1p Mn^{2+} NOG	Tpa1p Mn^{2+} 2,4-PDCA	Tpa1p Mn^{2+} IOX3
X-Ray source	Diamond Light Source beamline I04	Diamond Light Source beamline I04-1	In-house	Diamond Light Source beamline I04	Diamond Light Source beamline I04
Wavelength (Å)	0.83440	0.97949	1.5418	0.83440	1.2716
PDB acquisition code	4NHX	4NHY	4NHL	4NHK	4NHM
Resolution[a] (Å)	45.2–2.10 (2.18–2.10)	48.2–2.60 (2.69–2.60)	29.7–2.84 (2.94–2.84)	48.6–1.90 (1.97–1.90)	46.9–1.90 (1.97–1.90)
Space group	$P3_221$	$P2_12_12$	$C2$	$C2$	$C2$
Unit Cell Dimensions					
a, *b*, *c* (Å)	64.4, 64.4, 232.0	108.7, 130.5, 175.8	168.2, 67.3, 71.0	168.0, 67.7, 70.9	169.4, 67.6, 71. 5
α, β, γ (°)	90, 90, 120	90, 90, 90	90, 105.1, 90	90, 104.9, 90	90, 105.3, 90
Molecules per ASU	1	4	1	1	1
Wilson *B* factor ($Å^2$)	43.8	42.3	44.5	35.3	34.6
Total no. of reflections observed	536,556	419,736	68,118	396,484	404,304
No. of unique reflections[a]	33,097 (2,981)	76,983 (7,587)	18,332 (1,806)	59,886 (5,937)	61,321 (6,037)
Multiplicity[a]	16.2 (6.1)	5.5 (5.5)	3.7 (3.7)	6.6 (5.9)	6.6 (6.3)
Completeness[a] (%)	99.1 (91.7)	100.0 (100.0)	100.0 (100.0)	99.0 (98.2)	99.4 (98.5)
$I/\sigma(I)$[a]	17.4 (2.5)	12.7 (1.9)	7.4 (2.1)	24.7 (2.5)	26.5 (2.3)
[b]R_{cryst}	0.1887	0.1854	0.1810	0.1546	0.1449
[c]R_{free}	0.2154 (6.1)	0.2278 (2.6)	0.2425 (10.0)	0.1758 (3.3)	0.1704 (3.3)
Deviation from Idealized Geometry					
Bond lengths (Å)	0.006	0.007	0.011	0.010	0.010
Bond angles (°)	1.0	0.9	1.3	1.2	1.3
Average *B* factor[d] ($Å^2$)					
All atoms	50.8 (3,970)	61.1 (15,237)	42.7 (4,467)	44.8 (4,776)	42.4 (4,974)
Protein	50.6 (3,777)	61.2 (15,084)	42.9 (4,382)	44.6 (4,380)	41.5 (4,407)
Inhibitor	35.2 (10)	50.7 (48)	33.0 (10)	34.5 (12)	35.1 (19)
Metal (Mn^{2+})	31.9 (1)	47.4 (4)	34.3 (1)	28.3 (1)	23.5 (1)
Water	54.9 (175)	43.7 (83)	31.4 (74)	46.2 (359)	49.7 (535)
Ramachandran Plot					
Favored (%)	96.7	96.0	95.7	98.0	98.3
Allowed (%)	3.3	4.0	4.3	2.0	1.7
Disallowed (%)	0	0	0	0	0

[a]High-resolution shell in parentheses.
[b]$R_{cryst} = \sum||F_{obs}| - |F_{calc}||/|F_{obs}|$.
[c]Percentage of the total reflections used for R_{free} calculation in parentheses.
[d]Numbers of atoms in parentheses.

approximately half of the proposed substrate binding groove near the active site (see below). A cluster of five helices ($3_{10}1$, $\alpha1$–$\alpha4$) buttress the major β sheet of the NTD.

The CTD of OGFOD1 lacks a metal binding site and contains nine helices ($3_{10}3$–$3_{10}6$, $\alpha5$–$\alpha9$) and 11 β strands (β14–β24) (Figures 2 and S1A). Seven antiparallel β strands (β14-β22(βVI′)-β19(βIII′)-β24(βVIII′)-β17(βI′)-β16-β15) form the major β sheet, and four antiparallel β-strands (β18(βII′)-β23(βVII′)-β20(βIV′)-β21(βV′)) form the minor β sheet at the CTD DSBH core. In addition, a cluster of six helices ($3_{10}3$, $3_{10}4$, $\alpha6$–$\alpha9$) buttress the major β sheet of the CTD. Despite the common DSBH fold, the NTD and CTD display relatively low sequence and structural similarity to each other (sequence identity 12%; root-mean-square deviation [rmsd] value of 2.6 Å over 187 C^{α} atoms).

Comparison of OGFOD1 and Tpa1p

Superimposition of the OGFOD1 and Tpa1p structures reveals that the NTDs are well conserved (sequence identity 33%; rmsd 1.8 Å over 201 C^{α} atoms) (Figure 3). At least in the crystalline state, the CTD of OGFOD1 appears to be more compact than that of Tpa1p (sequence identity 23%; rmsd 2.0 Å over 186 C^{α} atoms). OGFOD1 also has a shorter loop ($313–318_{OGFOD1}$) linking $\alpha7$ and the β15-β16 hairpin corresponding to $383–402_{Tpa1p}$. The helices and loops ($417–470_{Tpa1p}$) between $3_{10}4$ and $\alpha8$ and the extension ($518–532_{Tpa1p}$) between the β strands βII′(β18) and βIII′(β19) in the CTD DSBH of Tpa1p are absent from OGFOD1 (Figures 3 and 4).

The CTD of OGFOD1 differs from that of Tpa1p by the presence of an additional 3_{10} helix, $3_{10}6$ ($518–531_{OGFOD1}$) that links

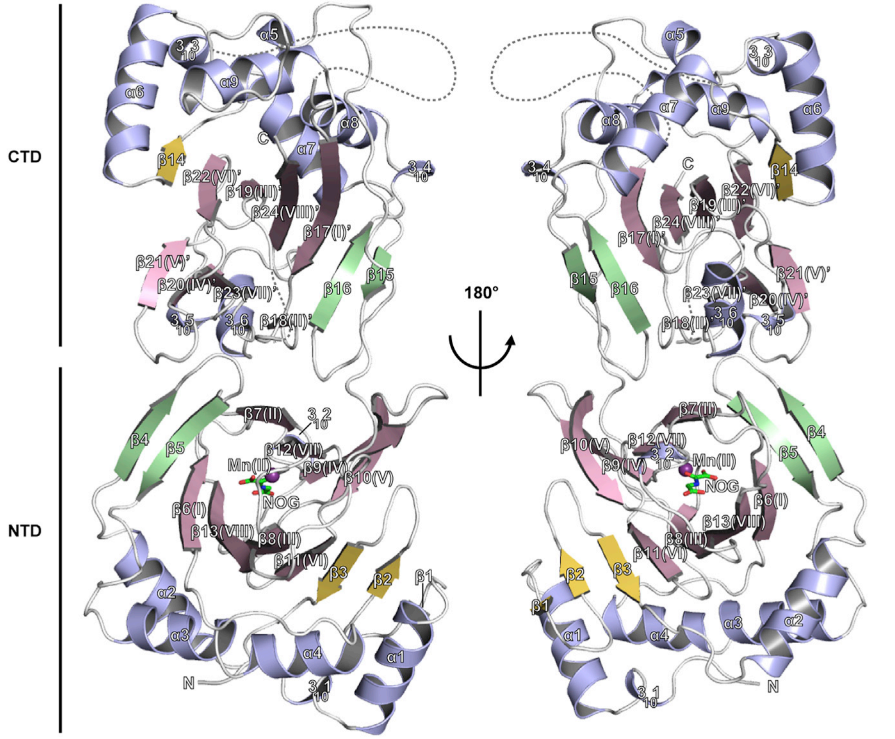

Figure 2. Ribbon Representations Showing an Overall View of the OGFOD1 Crystal Structure

Coloring scheme: α helices and 3_{10} helices (blue), β strands forming the core double-stranded β-helix (DSBH) fold (pink), β strands forming the β4-β5 hairpin (green), and all other strands (yellow). The DSBH β strands are additionally labeled with Roman numerals as in Clifton et al. (2006). A hypothetical position of the disordered acidic region (60 residues) linking α9 and β17 in the OGFOD1 C-terminal domain (CTD) is represented by a dashed line.

β23 and β24, and an "acidic" disordered region ($371–430_{OGFOD1}$) of unknown function that is not observed in the OGFOD1 electron density maps (Figures 2 and 3). The CTD helices ($3_{10}3$–$3_{10}5$, α6–α9) that buttress the major β sheet are structurally conserved in both OGFOD1 and Tpa1p. In general, the catalytic NTDs of OGFOD1 and Tpa1p are very similar, but the CTDs are less so, possibly highlighting differences in regulatory mechanisms that may exist between the human and yeast uS12 hydroxylases (Lee et al., 2009; Yeh et al., 2011; Katz et al., 2014; Loenarz et al., 2014; Singleton et al., 2014; Figure 4).

There are clear structural differences between the NTD-CTD linker regions of OGFOD1 and Tpa1p (Figure 3). In OGFOD1, the NTD-CTD linker region comprises 31 residues (239–269), eight of which are prolines, and has loop secondary structure. The high proline residue content apparently serves to rigidify the linker conformation. The linker region in Tpa1p is longer than in OGFOD1, comprising 95 residues (247–341) with four α helices (residues 259–266, 269–277, 294–304, and 332–339) and one 3_{10} helix (279–282), and low proline content. In OGFOD1, the buried surface area between the NTD and CTD is ~700 $Å^2$, and involves four hydrogen bonds and two salt bridges. In contrast, in Tpa1p the buried surface area is ~1000 $Å^2$, with 17 hydrogen bonds and four salt bridges (excluding the NTD to CTD linker region). Despite the presence of more intramolecular interactions at the domain interface, there is no substantial difference in the relative positions of the NTD and CTD in OGFOD1 and Tpa1p structures (sequence identity 27%; rmsd 2.5 Å over 408 C^{α} atoms).

Previous structural studies on Tpa1p, which is reported to be a homodimer in solution, identified a large dimerization interface between the CTDs of two protomers (buried surface area ~1900 $Å^2$) (Kim et al., 2009; Henri et al., 2010; Figure 3B). In contrast, the OGFOD1 structure does not share an intermolecular interface in this region despite having a crystal packing arrangement similar to that of Tpa1p (Figure 3). This observation correlates with our native PAGE results showing that OGFOD1 does not form oligomers, whereas Tpa1p forms a dimer under non-denaturing conditions (Figure 5H). The observed Tpa1p CTD dimerization interface comprises structural elements that are absent from OGFOD1, including the elongated NTD-CTD linker region (Tpa1p residues 247–341, 417–470, and 518–532) (Figures 3 and 4).

Structurally informed sequence comparison reveals that the *S. pombe* Ofd1 has, like OGFOD1, a relatively short NTD-CTD linker sequence, and that structural dimerization elements observed in Tpa1p are absent from Ofd1 (Figure 4). Sequence analysis indicates that the loop linking the β strands β(II′) and β(III′) in Ofd1 and OGFOD1 ($414–420_{Ofd1}$, $450–458_{OGFOD1}$, $518–532_{Tpa1p}$) is shorter than in Tpa1p, where it is involved in dimerization (Kim et al., 2009; Figure 4). Thus, overall Ofd1 is more like OGFOD1 than Tpa1p in the observed Tpa1p dimerization regions.

OGFOD1 and Tpa1p Active Site and Inhibitor Binding

The active site of OGFOD1 is similar to that of Tpa1p (Figures 5A–5E). The OGFOD1 active site contains an HXD…H metal binding facial triad (residues His155, Asp157, and His218), the side chains of which octahedrally coordinate the metal along with the inhibitor and a water molecule (W1, Figure 5A). His155 and Asp157 are positioned on β strand βII(β7) and the loop region between β strands βII(β7) and βIII(β8), respectively; the "distal" metal binding histidine, His218, originates from β strand βVII(β12). In all of the OGFOD1 and Tpa1p structures, the inhibitors ligate the metal in a bidentate manner (Figures 5A–5E).

OGFOD1 and Tpa1p bind NOG similarly. With OGFOD1 the C-5 carboxylate of NOG is positioned to form hydrogen bonds to the side chains of Tyr169 and Arg230 (analogous interactions occur with Tpa1p); the latter is part of a conserved RXS motif that binds to the 2OG C-5 carboxylate in a large subfamily of 2OG oxygenases (e.g. deacetoxycephalosporin C synthase) (Ser232 Oγ and NOG C-5 carboxylate oxygen distance is 4.4 Å) (Clifton et al., 2006; Aik et al., 2012; Figures 5A and 6A). A glycerol molecule is observed in the apparent OGFOD1 substrate binding site

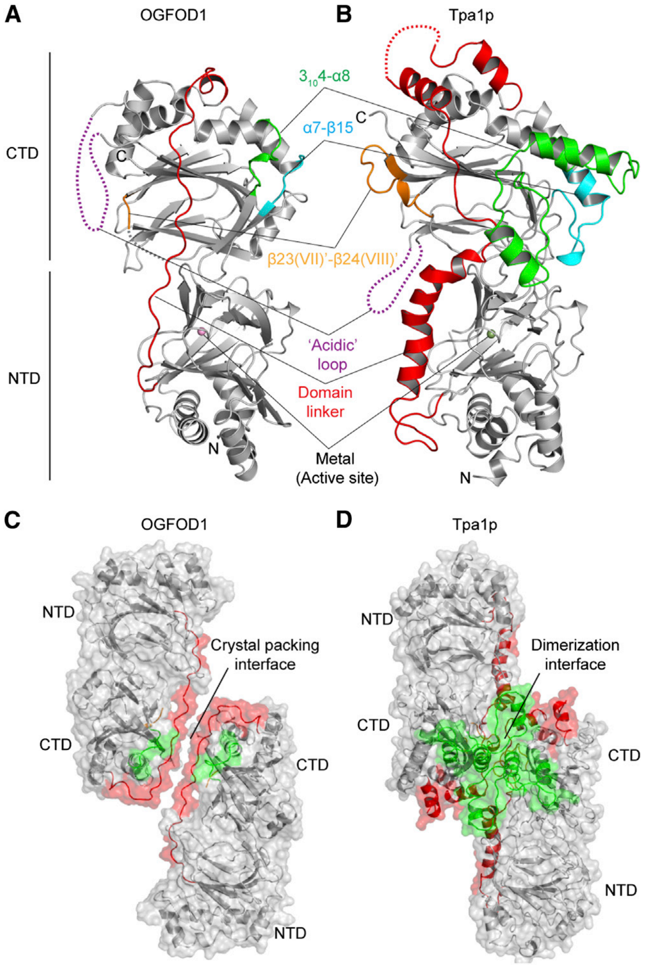

Figure 3. Structural Comparison of OGFOD1 and Tpa1p

(A and B) Comparison of OGFOD1 (PDB ID: 4NHX) (A) and Tpa1p (PDB ID: 3KT4) (B) (Kim et al., 2009) structures reveals differences in the linker regions in OGFOD1 (residues 239–269) and Tpa1p (residues 247–341) (red).

(C and D) The Tpa1p dimer interface (D) includes residues 383–402 (cyan), 417–470 (green), and 518–532 (yellow), corresponding to OGFOD1 residues 313–318, 332–339, and 456–458, respectively (C).

and forms a hydrogen bond to the NOG oxalyl carboxylate (2.3 Å).

The OGFOD1:2,4-PDCA structure reveals slight variations in the inhibitor hydrogen bonding patterns between the four chains in the ASU, suggesting flexibility within the 2OG binding pocket (Figures 5G and 6B). OGFOD1 and Tpa1p bind 2,4-PDCA slightly differently (Figure 5F). In the Tpa1p:Mn(II):2,4-PDCA structure the 2,4-PDCA C-4 carboxylate is positioned to form a hydrogen bond to the side chain hydroxyl of Ser240, yet surprisingly is not positioned to form a salt bridge to the side chains of Arg238 or Tyr173 (corresponding to Arg230/Tyr169$_{OGFOD1}$), respectively; in part this appears to be due to the unusual conformation of Arg238. These observations provide further evidence of flexibility within the 2OG binding pocket of the uS12 hydroxylases, a feature that might be exploited in selective inhibitor design.

In Tpa1p, IOX3 binds similarly to NOG with its glycyl side chain carboxylate positioned to form a salt bridge with the Arg238 side chain (3.1 Å) (Figure 5E) and a hydrogen bond to the Tyr173 hydroxyl (2.6 Å). The Tyr150 hydroxyl forms a hydrogen bond (2.8 Å) to the IOX3 bicyclic ring C-9 hydroxyl (Figure 5E). All the residues of Tpa1p that interact with IOX3 are conserved in OGFOD1, suggesting that IOX3 will bind to the 2OG binding pocket of OGFOD1 in the same way.

For OGFOD1 the IC_{50} value of IOX3 inhibition was 520 ± 80 nM, which is comparable with the value for PHD2 inhibition (IC_{50} 1.4 μM), and lower than the value for several histone lysine demethylases (KDMs) (IC_{50} >20μM) (Chowdhury et al., 2013; Figure 6C). Comparison of the Tpa1p and PHD2 structures in complex with IOX3 reveals highly similar binding modes reflecting the general conservation in the 2OG binding pocket of PHs (Chowdhury et al., 2009; Figures 7B, 7G and 7H). Thus, IOX3 along with related inhibitors are likely relatively potent inhibitors of both OGFOD1 and PHD2 (PHD2 is the most important PHD in the human hypoxic response), information that may be relevant to the interpretation of ongoing clinical trials with PHD inhibitors. However, in contrast to crystallographic results, nuclear magnetic resonance studies of an IOX3 analog binding to PHD2 indicated the presence of two different binding modes in solution (Poppe et al., 2009). Thus we cannot rule out the possibility of flexibility in the coordination mode to OGFOD1 in solution. Differences in the identity of residues positioned near the active site opening (Tpa1p/PHD2: Leu156/Tyr310, Gln242/Thr387) may permit the design of selective inhibitors by substitutions at positions between C-1 to C-6 and C-10 of the IOX3 heteroaromatic scaffold or by similar modifications to other 2OG competitive inhibitors. In both Tpa1p and PHD2, IOX3 forms an internal hydrogen bond between its C-9 hydroxyl and N-15 amide, likely contributing to rigidity and promoting inhibition.

Active Site Comparison of OGFOD1 with Other PHs

The metal and NOG in the OGFOD1 active site are positioned similarly to those in the PHD2 active site (Figures 4 and 8). With PHD2, one of the NTD metal ligands, i.e. the Asp315 carbonyl oxygen, is also positioned to hydrogen bond with a metal-bound water (W1); this interaction is proposed to stabilize the interaction between the water and metal, thus increasing the ability of water to compete with O_2 for Fe(II) binding and rendering O_2 binding to PHD2 generally slow, a property proposed to be relevant to its hypoxia sensing role (Neidig et al., 2007; Flashman et al., 2010; Flagg et al., 2012; Figure 8C). Analysis of the active site water molecules in the vicinity of the metal also reveals similarity between OGFOD1 and PHD2 (McDonough et al., 2006; Figures 8A and 8C). Like PHD2, OGFOD1 has a "second shell" water (W2) (second shell in relation to metal binding) positioned to hydrogen bond with the metal-bound water (W1) and the amide carbonyl of Gly235$_{OGFOD1}$ (in PHD2, the Thr387$_{PHD2}$ hydroxyl apparently plays an analogous role). However, in the available OGFOD1 structures a distinct "third shell" water (W3) is observed at the active site that forms a hydrogen bond to the hydroxyl of Ser234$_{OGFOD1}$ (Figure 8A); the electron density maps, distance, and geometry indicate that W3 is weakly bound. Compared with OGFOD1 and PHD2, the structures of other 2OG oxygenases shown in Figure 8 (including Factor-inhibiting HIF [FIH], which is active at lower O_2 levels than the PHDs [Koivunen et al., 2004; Tian et al., 2011]) lack or show weaker first and second shell active site water interactions,

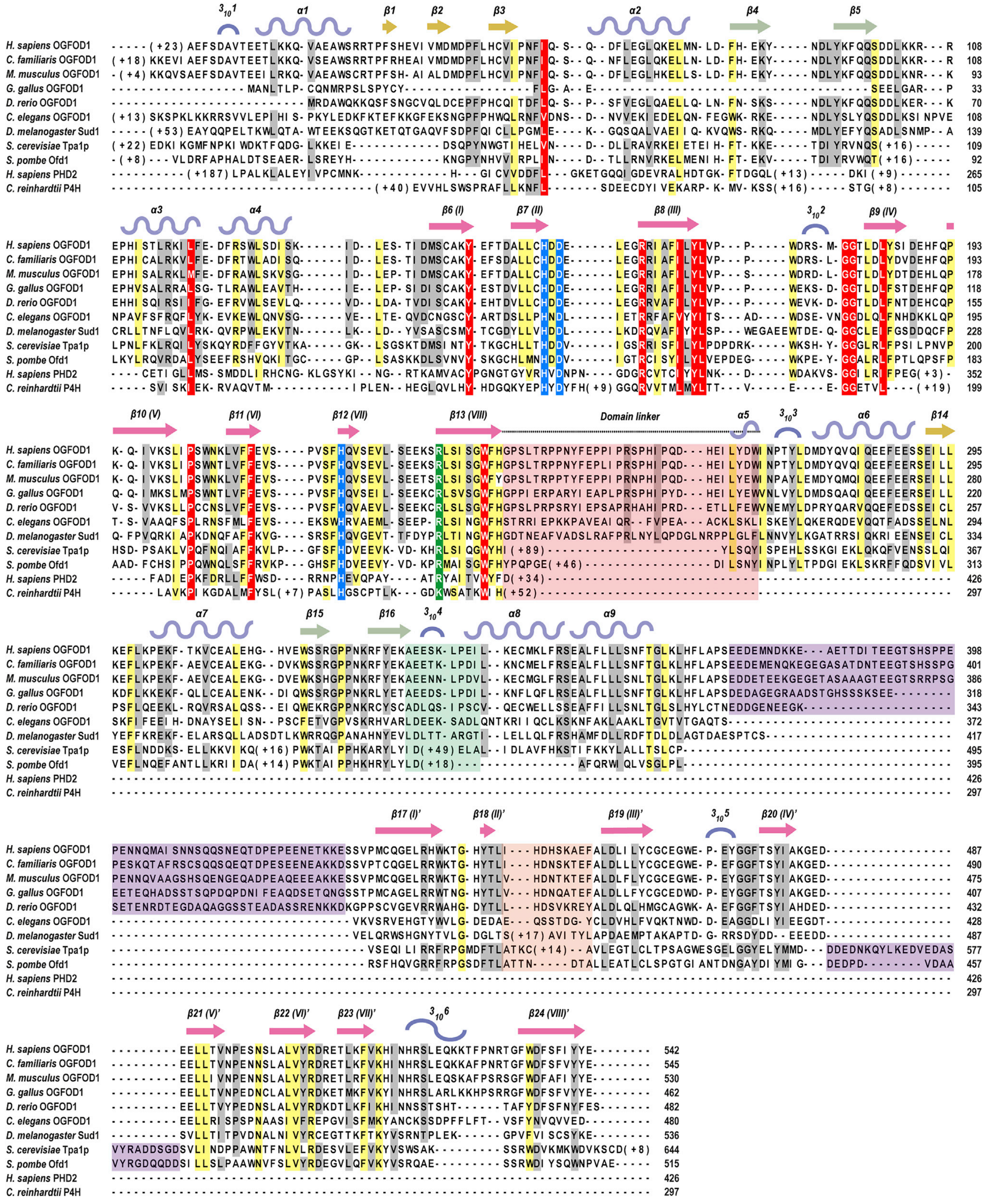

Figure 4. Structure-Based Sequence Alignment

Structure-based sequence alignment of *Homo sapiens* OGFOD1 (GI 94536836), *Saccharomyces cerevisiae* Tpa1p (GI 731462), *Homo sapiens* PHD2 (GI 32129514), and *Chlamydomonas reinhardtii* P4H (GI 159478673) (STRAP) (Gile and Frömmel, 2001). Clustal W-generated (Larkin et al., 2007) sequence alignment

(legend continued on next page)

consistent with the proposal that in the case of PHD2 and perhaps the other PHDs and OGFOD1, metal solvation by water slows the reaction with oxygen (Figure 8).

Ofd1 activity in cells is affected by O_2 levels and is proposed to act as a hypoxia sensor (Lee et al., 2009; Loenarz et al., 2014), although the mechanism of its O_2-dependent role is unclear. Ofd1 is similar to OGFOD1 with respect to the presence of a serine residue ($Ser225_{Ofd1}$) at the same position as the W3 coordinating $Ser234_{OGFOD1}$. However, in Tpa1p, where no W3 is observed, the corresponding residue is a glutamate ($Glu242_{Tpa1p}$), which may reflect the relatively lower O_2 sensitivity observed for Tpa1p activity in cells (Loenarz et al., 2014). In PHD2 the equivalent residue is $Thr387_{PHD2}$, but this residue is not observed to bind to a third shell water. Instead the $Thr387_{PHD2}$ hydroxyl directly interacts with a second shell water W2 (Figure 8).

Substrate Binding Groove Comparison of OGFOD1/Tpa1p/PHD2/CrP4H

Superimposition of the NTD structure of OGFOD1 with the structures of PHD2 in complex with HIF substrate, and a collagen-like P4H from *C. reinhardtii* (CrP4H) in complex with a proline-rich substrate, enabled the identification of a substrate binding groove in OGFOD1 lined by residues Trp236, Arg162, Leu152, Asp140, Gln100, Tyr96, Lys91, Leu95, Leu159, and Asp156 (McDonough et al., 2006; Koski et al., 2007; Chowdhury et al., 2009; Koski et al., 2009; Figures 7C and 7F). A more global analysis of conserved residues using ConSurf, which projects a color-based residue conservation level among homologous protein sequences onto the structure (Landau et al., 2005), implies high conservation in and around the immediate vicinity of the proposed OGFOD1 substrate binding groove (Figure 9). Studies employing variants in yeast cells support the proposed substrate binding groove: OGFOD1 R162A exhibited partially reduced activity, and the variants L95A and Y96A from the β4-β5 hairpin, and L152Y from β(II), produced to make OGFOD1 more PHD-like, all showed significantly reduced activity (Figures 7I and S2B).

The OGFOD1 β4-β5 hairpin (14 residues, 88–101) is in the equivalent region of the mechanistically important β2-β3 finger loop of the P4Hs, PHD2 (23 residues, 235–257) and CrP4H (25 residues, 74–98) (Chowdhury et al., 2009; Koski et al., 2009; Figures 7A and 7D). The flexible β2-β3 finger loop forms a lid that acts to enclose and position the substrate for *trans*-4 prolyl hydroxylation (Chowdhury et al., 2009; Koski et al., 2009). Structures of PHD2 and CrP4H show the β2-β3 finger loop "closed" in their substrate-bound states and partially disordered in structures without substrate, demonstrating this region's flexibility and supporting an induced-fit mechanism (Chowdhury et al., 2009; Koski et al., 2009). In contrast, the β4-β5 hairpins of OGFOD1 and Tpa1p are involved in interactions at the NTD/CTD interface. Contrary to the flexible disordered β2-β3 finger loops in the PHD2/CrP4H structures without substrate, there is well-defined electron density observed for the relatively short β4-β5 hairpins of OGFOD1 and Tpa1p (14 residues, 80–93) without substrate. These observations suggest that the uS12 hydroxylases do not utilize their short and rigid β4-β5 hairpin fingers in the same induced-fit mechanism as do PHD2 and CrP4H (Chowdhury et al., 2009; Koski et al., 2009). The C-terminal regions of PHD2 and CrP4H interact with their respective substrates, are flexible, and are also involved in the induced-fit mechanism (Chowdhury et al., 2009; Koski et al., 2009). The equivalent regions of OGFOD1 and Tpa1p catalytic NTD form part of the rigid NTD-CTD linker and are unlikely to play a role similar to that in the P4Hs.

We tested whether a disordered "acidic region" of OGFOD1 ($371–430_{OGFOD1}$, with a calculated net charge of −14 at neutral pH) between α9 and β17 in the CTD might play a role in binding the highly basic uS12 substrate (Chowdhury et al., 2009; Koski et al., 2009). We generated OGFOD1 and Tpa1p variants lacking acidic loop regions near the active site ($\Delta374–426_{OGFOD1}$ and $\Delta561–584_{Tpa1p}$). Both variants retained uS12 hydroxylation activity in yeast cells, indicating that the acidic regions are not essential for catalytic activity. Although a catalytic role for the acidic region seems unlikely, it may contribute to roles in protein-protein interactions, e.g. Nro1 (negative regulator of Ofd1) and Ett1 (Nro1 ortholog) for Tpa1p, and/or protein-nucleic acid interactions (Lee et al., 2009). Tpa1p does not have an acidic region in the same region as OGFOD1, but does have a disordered acidic region ($561–586_{Tpa1p}$, calculated net charge −8 at neutral pH), corresponding to a shorter, less acidic, and more ordered loop between βIV′(β20)-βV′(β21) in OGFOD1 ($484–488_{OGFOD1}$, with a calculated net charge of −2 at neutral pH) positioned near the periphery of the active site (Figures 3 and 4), which may be involved in substrate binding (Ofd1 and Tpa1p are similar in this regard) (Figure 4).

DISCUSSION

The uS12 ribosomal hydroxylases are the only 2OG oxygenases so far identified with tandem DSBH domains. However, tandem DSBH-containing proteins that are not 2OG dependent include the human transcriptional cofactor protein Pirin (PDB ID: 1J1L), *Bacillus subtilis* oxalate decarboxylase (OXDC; PDB ID: 1J58) and *Aspergillus japonicus* quercetin 2,3-dioxygenase (2,3QD; PDB ID: 1H1I) (Anand et al., 2002; Steiner et al., 2002; Pang et al., 2004). A notable structural difference between OGFOD1/Tpa1p and the 2OG independent tandem DSBH proteins is that the two domains of OGFOD1/Tpa1p pack against each other via their minor β sheet, whereas for Pirin and related tandem DSBH structures the two domains pack against each other via their major β sheet (Figure S1). The CTDs of OGFOD1, Tpa1p, Pirin, and 2,3QD do not have metal ion binding sites while OXDC has metal binding sites in both the NTD and CTD (Anand et al., 2002). These major differences suggest that the OGFOD1 subfamily emerged independently rather than from the other tandem DSBH proteins mentioned above.

of OGFOD1 and homologs from higher eukaryotes, *Canis familiaris* (GI 73949826), *Mus musculus* (GI 34850072), *Gallus gallus* (GI 118096214), *Danio rerio* (GI 41054417), *Caenorhabditis elegans* (GI 17531931), *Drosophila melanogaster* (GI 74942745), and *Schizosaccharomyces pombe* (GI 2894283). Strictly conserved residues are shown in red, highly conserved residues in yellow, semi-conserved residues in gray, the conserved metal binding triad in blue, and residue that binds the 2OG C-5 carboxylate in green. Boxed regions represent the disordered acidic loops in OGFOD1 (light green; residues 371–430) and Tpa1p (light blue; residues 561–586), and the proposed dimerization interface (red/green/orange).

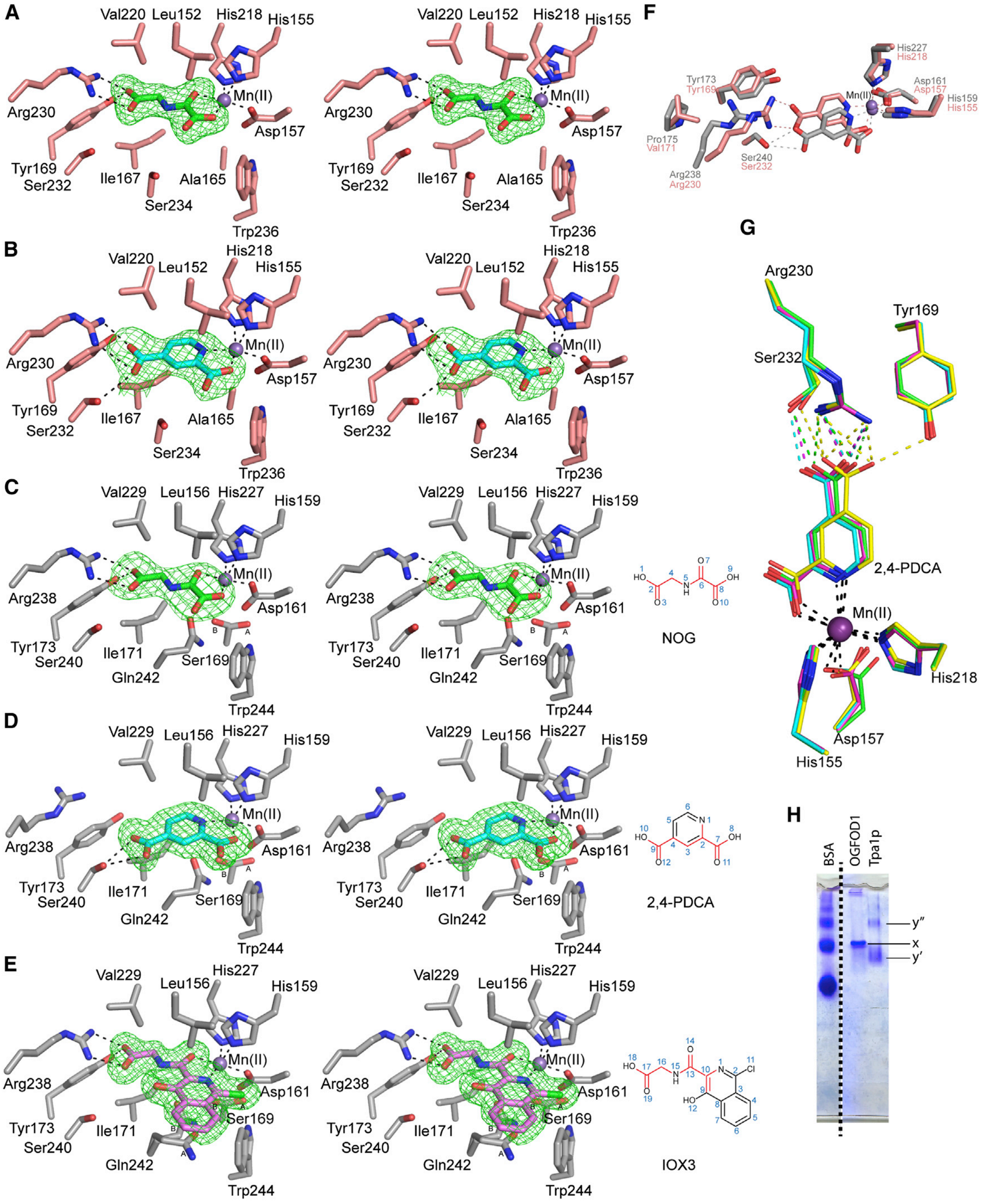

Figure 5. Wall-Eyed Stereoviews of the Active Sites of OGFOD1/Tpa1p Inhibition Complexes

(A–E) OGFOD1:NOG (A), OGFOD1:2,4-PDCA (B), Tpa1p:NOG (C), Tpa1p:2,4-PDCA (D), and Tpa1p:IOX3 (E). The electron density maps OMIT $|mF_o - DF_c|$ (shown in green mesh) are contoured to 3.0σ.

(F) Superimposition of OGFOD1:2,4-PDCA (chain A) (pink) and Tpa1p:2,4-PDCA (silver) active site views reveal different binding modes for 2,4-PDCA and conformations of $Arg230_{OGFOD1}$/$Arg238_{Tpa1p}$.

(G) Superimposition of OGFOD1 chains A (green), B (cyan), C (magenta), and D (yellow) in complex with 2,4-PDCA. Active site residues are shown as sticks and Mn(II) as a sphere (purple).

(H) Non-denaturing PAGE analysis of OGFOD1 and Tpa1p. x, y′ represent monomers; y″ is proposed to be the Tpa1p dimer.

Bioinformatics analysis indicates that most, if not all, prokaryotic prolyl and proline hydroxylases are single-domain enzymes (Clifton et al., 2001; Koketsu et al., 2014; Scotti et al., 2014). The OGFOD1 subfamily PHs are found only in eukaryotes and may have branched off from other single DSBH domain PHs during evolution from a common ancestor, possibly by a gene

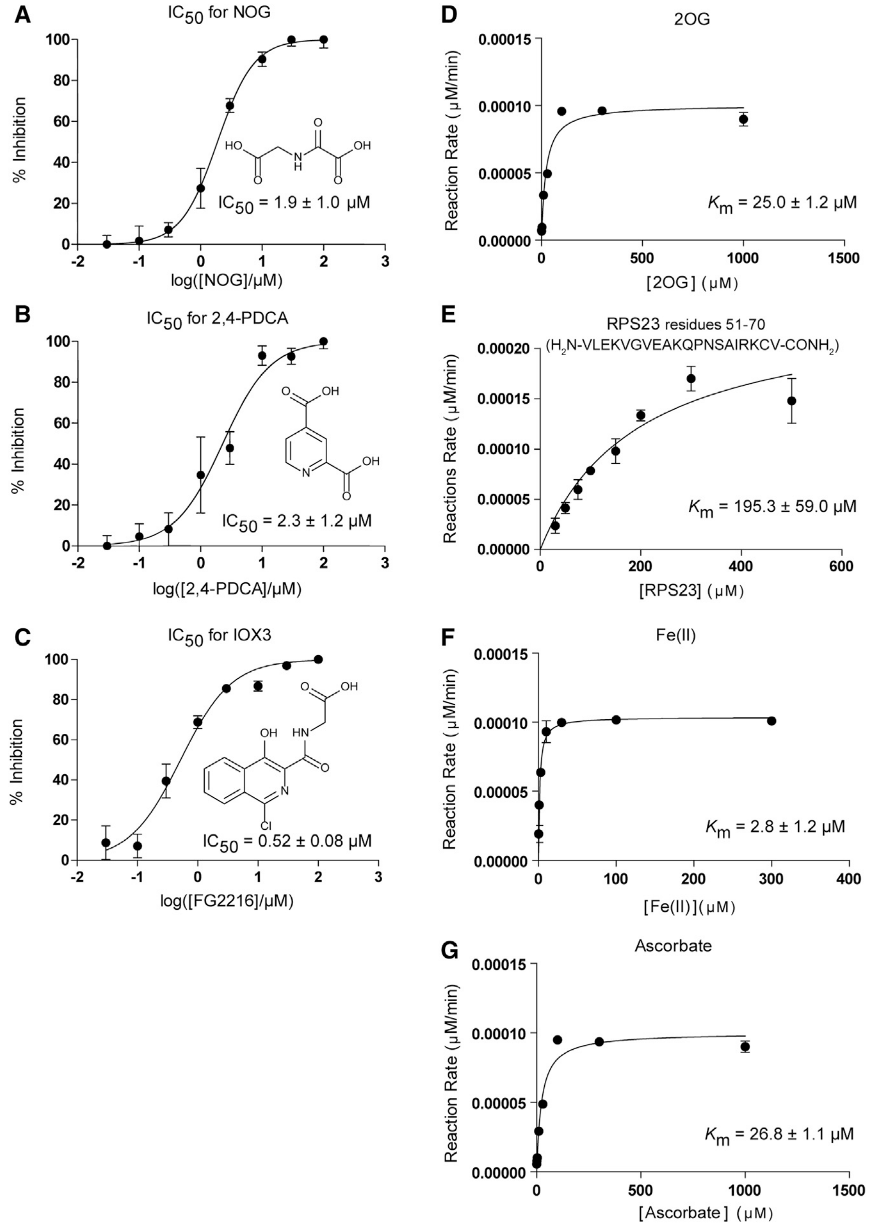

Figure 6. Kinetic Characterization of OGFOD1

(A–C) Kinetic characterization of OGFOD1 toward NOG (A), 2,4-PDCA (B), and IOX3 (C). The data points represent the average, and error bars the SD of triplicate samples.

(D–G) The concentrations of the assay conditions, optimized based on the K_m values of 2OG (D), RPS23$_{51-70}$ (E), Fe(II) (F), and ascorbate (G).

duplication event (Scotti et al., 2014). Having lost catalytic potential due to redundancy, the CTD may have retained or gained function(s) in substrate and/or binding partner recognition, stabilization, and/or oligomerization. A role for the non-catalytic CTD in the OGFOD1 subfamily in binding to the uS12 substrate, or other binding partners, seems likely; it is reported that the CTDs of both Ofd1 and Tpa1p interact with Nro1 and Ett1, respectively (Lee et al., 2009; Rispal et al., 2011). Non-DSBH domains have been shown to be important in oligomerization and substrate binding selectivity in the case of other 2OG oxygenases, e.g. in some *N*-methyl lysine demethylases (KDMs) (Horton et al., 2010). Other 2OG oxygenases, such as the collagen PHs CPH and Leprecans, have an all helical repeat domain, tetratricopeptide repeat (TPR), that functions in substrate recognition (Pekkala et al., 2004; Koski et al., 2009). TPR domains are also found in the epidermal growth factor-like domain aspartyl/asparaginyl hydroxylase and the KDMs UTX and UTY (ubiquitously transcribed X and Y chromosome TPR protein, respectively) (Pollard et al., 2008; Chowdhury et al., 2014). However, less is known of the functional roles of the additional domains in these cases. It is possible that the helical repeat protein Nro1/Ett1 acts as a substrate recognition module for Tpa1p and/or Ofd1 through its interaction with the CTD in a similar manner to the TPR fused to the PH domain, which would explain why in vitro activity on uS12 peptide fragments using isolated recombinant Tpa1p has not been observed. A C-terminal winged helix domain of unknown function has been observed in the homodimer ROXs (MINA53, NO66, and YcfD) (Chowdhury et al., 2014); this may be involved in substrate recognition and/or other protein-protein interactions, as proposed for the CTD of the OGFOD1 subfamily.

The results reveal a high degree of structural conservation between the catalytic domain of PHDs and OGFOD1/Tpa1p, consistent with a common ancestry. Although the precise biochemistry underlying their different selectivities will require further investigations, examination of the available PH structures provides potential insight into how stereo- and regioselective hydroxylation is achieved. All P4Hs and P3Hs have a conserved arginine (Arg322$_{PHD2}$, Arg161$_{CrP4H}$) at their active site opening (Figure 4), which is positioned to form a hydrogen bond to the backbone carbonyl oxygen of the substrate proline, as has been observed for PHD2, CrP4H, and PPHD (Figures 7B, 7E, and 7H). All P4Hs additionally share a conserved tyrosine (Tyr310$_{PHD2}$; Tyr140$_{CrP4H}$), the side chain hydroxyl of which points toward the amide nitrogen of the substrate prolyl residues. These residues apparently help to maintain the substrate in position for stereoselective (*trans*-) and regioselective (C-4) hydroxylation (Scotti et al., 2014; Figure 4). In contrast, P3Hs have a leucine residue at the corresponding position (Leu152$_{OGFOD1}$; Leu156$_{Tpa1p}$), which may favor prolyl 3-, over prolyl 4-, hydroxylation by shifting binding of the substrate backbone with respect to the metal.

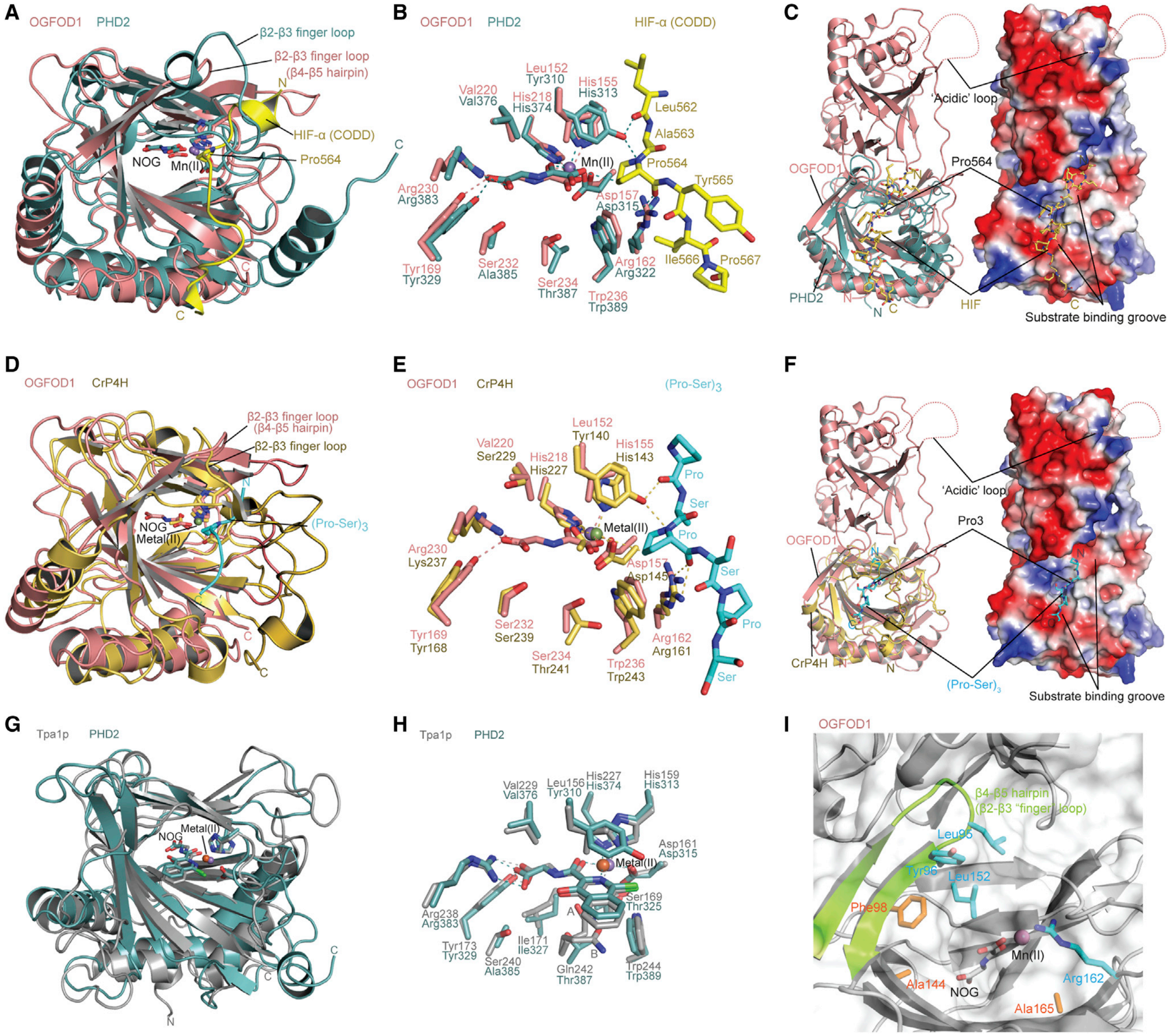

Figure 7. Comparison of Structures of Human P3H and P4H: OGFOD1/Tpa1p and PHD2/CrP4H, respectively

The coloring scheme is as in other figures. Active site residues are displayed as sticks and Mn as a sphere.

(A and B) Overall structure (A) and active site (B) of OGFOD1:NOG (PDB ID: 4NHX) (pink) superimposed onto the catalytic domain of human PHD2 (PDB ID: 3HQR) (green) in complex with an HIF-1α C-terminal oxygen-dependent degradation domain (CODD) fragment (558–574) (yellow).

(C) Ribbons and electrostatic surface representations of the OGFOD1:Mn:NOG structure (pink/surface) superimposed on that of PHD2 (green) in complex with the HIF-1α CODD (yellow) (Chowdhury et al., 2009), highlighting the putative OGFOD1 substrate-binding groove.

(D and E) Overall (D) and active site (E) superimpositions of OGFOD1:NOG (PDB ID: 4NHX) (pink) and *C. reinhardtii* P4H (Koski et al., 2009) (PDB ID: 3GZE) (pale yellow) in complex with substrate peptide (cyan).

(F) Ribbons and surface representations of the OGFOD1:Mn:NOG structure (PDB ID: 4NHX) (pink/surface) superimposed on a *C. reinhardtii* P4H structure (Koski et al., 2009) (PDB ID: 3GZE) (pale yellow) in complex with its peptide substrate (cyan), highlighting the putative substrate-binding groove of OGFOD1.

(G and H) Overall (G) and active site (H) superimpositions of Tpa1p:IOX3 (PDB ID: 4NHM) (gray) and PHD2 (Chowdhury et al., 2013) (PDB ID: 4BQY) (green) in complex with inhibitors.

(I) Active site view of OGFOD1 (PDB ID: 4NHX) with ribbons and transparent surface. β4-β5 hairpin and the residues with which variants were made are shown as ribbons (green) and stick models (cyan/orange). The residues in sticks are those playing critical (cyan) and no (orange) apparent roles in catalysis as shown by cellular studies.

However, substitution of Tyr152 of OGFOD1 for a leucine residue ablated activity in cells within limits of detection, indicating that this residue alone does not determine C-3 versus C-4 regioselectivity (Figure 7I). Furthermore, the OGFOD1 active site is apparently more open than that of PHD2 (McDonough et al., 2006) and CrP4H (Koski et al., 2007), in part due to its shorter β4-β5 hairpin and lack of a C-terminal helix (which are important in substrate binding for the P4Hs) (Chowdhury et al., 2009; Koski et al.,

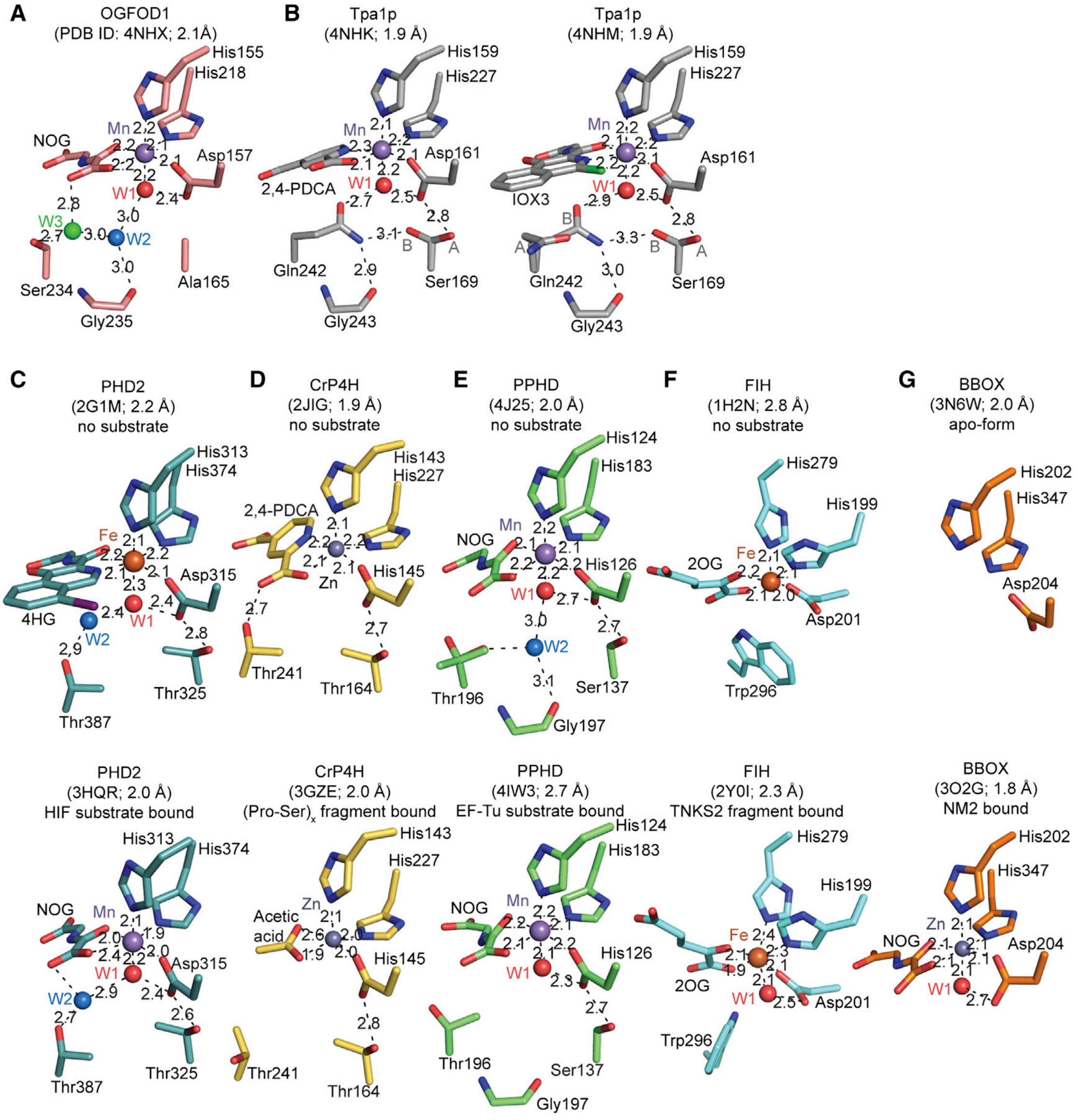

Protein Name	PDB ID	Distance (Å) Metal-W1	W1-W2	W2-W3
OGFOD1	4NHX	2.2	3.0	3.0
Tpa1p	4NHK	2.2	-	-
	4NHM	2.2	-	-
PHD2	2G1M	2.3	2.4	-
	3HQR	2.2	2.9	-
CrP4H	2JIG	-	-	-
	3GZE	-	-	-
PPHD	4J25	2.2	3.0	-
	4IW3	2.1	-	-
FIH	1H2N	-	-	-
	2Y0I	2.1	-	-
BBOX	3N6W	-	-	-
	3O2G	-	-	-

(legend on next page)

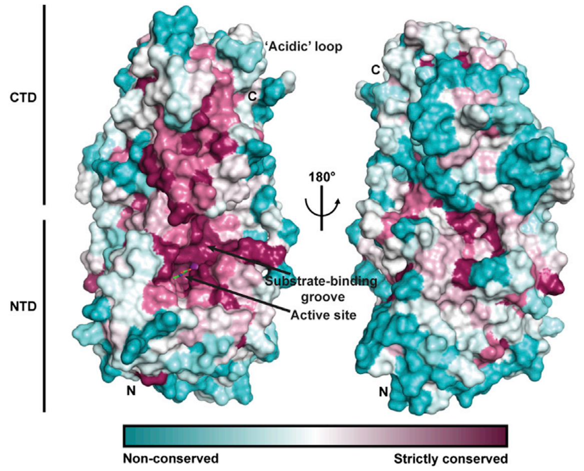

Figure 9. OGFOD1 ConSurf (Landau et al., 2005) Analysis Showing Residue Conservation

Non-conserved to strictly conserved residues are shown as a gradient from cyan to magenta, respectively. The most conserved region is located around the groove near the catalytic site in the NTD. Mn(II) (purple) and NOG (green) are shown. The active site is located in the middle of a groove formed by Lys91, Asp94, Leu95, Tyr96, Phe98, Gln100, Ser101, Asp103, Leu104, Lys105, Asp140, Ser142, Leu152, Asp156, Glu158, Leu159, Arg162, Trp236, Ile450, and Asp452. Lys91, Asp94, Leu95, Tyr96, Phe98, Gln100, and Ser101 are located on the β4-β5 hairpin region of the NTD; Asp103, Leu104, and Lys105 on the loop region between β5 and α3; Asp140 on the loop region between α4 and β6(βI); Ser142 and Leu152 on βI(β6) and βII(β7), respectively; Asp156, Glu158, and Leu159 on the loop region between βII(β7) and βIII(β8); Arg162 and Trp236 on βIII(β8) and βVIII(β13), respectively; Ile450 and Asp452 on the loop linking βII′(β18) and βIII′(β19).

2009). Importantly, comparison of the structures also reveals differences in and around the active site and substrate recognition elements, and in information regarding flexibility of residues forming the 2OG binding site, which will be useful for the identification of selective inhibitors for the HIF PHDs for which there are compounds presently in late stage clinical trials.

EXPERIMENTAL PROCEDURES

Protein Preparation and Purification

Escherichia coli BL21(DE3) cells were transformed with the pET28b_OGFOD1 (OGFOD1: 542 aa, 63 kDa) or pNIC28_Tpa1p (Tpa1p: 644 aa, 74 kDa) plasmids (encoding for proteins with an N-terminal hexa-histidine tag) (Loenarz et al., 2014) and grown (37°C; 180 rpm) to an OD_{600} of 0.6, after which recombinant protein production was induced using 0.5 mM β-D-1-thiogalactopyranoside (IPTG). The cells were then grown overnight at 18°C, harvested by centrifugation (10,000 × *g*; 7 min), and stored at −80°C for protein purification. Cell pellets were resuspended in a solution (100 ml) containing 50 mM HEPES (pH 7.5), 500 mM NaCl, and 20 mM imidazole, one EDTA-free protease inhibitor tablet (Roche), and approximately 1 mg DNaseI (bovine pancreas, grade II, Roche) at room temperature with gentle stirring. Cells were lysed on ice by sonication and the lysates were cleared by centrifugation (50,000 × *g*; 20 min). The supernatant was then purified with 5 ml of HisTrap Fast Flow column, which had been pre-equilibrated with the resuspension buffer, using an AKTA FPLC system (GE Healthcare). The column was washed with 50 mM HEPES (pH 7.5), 500 mM NaCl, and 50 mM imidazole, and the protein was eluted with 50 mM HEPES (pH 7.5), 500 mM NaCl, and 250 mM imidazole. The purified sample was then exchanged into 25 mM HEPES (pH 7.5) and 100 mM NaCl using a PD-10 column (Millipore). Following buffer exchange, 1 mM EDTA was added and the sample incubated for 3 hr at 4°C. The OGFOD1 or Tpa1p proteins were then further purified using a Superdex 200 size-exclusion column (GE Healthcare) that had been pre-equilibrated with 25 mM HEPES (pH 7.5) and 100 mM NaCl. Further purification was carried out by anion exchange chromatography using a 20-ml MonoQ column. Proteins were eluted using a gradient of 25 mM HEPES (pH 7.5) and 1 M NaCl. Protein-containing fractions were pooled, concentrated to 20 mg/ml by diafiltration and buffer exchanged into 10 mM Tris-HCl (pH 7.5), then aliquoted (20 μl), flash frozen in liquid N_2, and stored at −80°C.

OGFOD1 Activity Assays Inhibitor IC_{50} Determination

Concentrated OGFOD1 used for biochemical assays was stored at −80°C in 50 mM HEPES (pH 7.5), 150 mM NaCl, 1 mM DTT, and 5% (w/v) glycerol after HisTrap HP (5 ml) column purification. Activity assays were performed as reported (Loenarz et al., 2014), by determining the extent of hydroxylation of a 20-mer fragment of uS12 containing residues 51–70 (H_2N-VLEKVGVEAKQPNSAIRKCV-$CONH_2$) by MALDI-TOF mass spectrometry using a Waters Micromass MALDI micro MX mass spectrometer and MassLynx 4.1 software, as previously described (Flashman et al., 2008). The optimized hydroxylation assay involved incubation of OGFOD1 (1 μM) with inhibitor (1% v/v in DMSO) in the presence of Fe(II) (50 μM), 2OG (25 μM), ascorbate (100 μM), and $uS12_{51–70}$ (25 μM) in HEPES (50 mM, pH 7.5) at 37°C for 15 min. Reactions were quenched with formic acid (1% v/v). Samples were prepared by mixing the reaction mixture (1 μl) with α-cyano-4-hydroxycinnamic acid solution (water/acetonitrile 1:1) (1 μl). Dose-response studies employed point assays in triplicate; data were analyzed with GraphPad Prism 5.04.

Protein Crystallization and X-Ray Crystallography

Crystals of OGFOD1 in complex with NOG and 2,4-PDCA, and crystals of Tpa1p in complex with NOG, 2,4-PDCA, and IOX3 were grown by the sitting drop vapor diffusion method (drop size: 200–300 nl) at 293 K in 96-well Intelli-plates (Art Robbins). Crystals were cryo-protected by transfer to 25% glycerol in well solution and then harvested using nylon loops (Hampton Research), and cryo-cooled by plunging into liquid nitrogen. Data were collected at 100 K using single crystals at Diamond Light Source beamline I04 (OGFOD1:Mn(II):NOG, Tpa1p:Mn(II):2,4-PDCA, Tpa1p:Mn(II):IOX3) and Diamond Light Source beamline I04-1 (OGFOD1:Mn(II):2,4-PDCA). In-house data were collected at 100 K for single crystals of Tpa1p:Mn(II):NOG using a Rigaku FR-E+ Superbright copper rotating anode diffractometer equipped with Osmic HF optics and a Saturn 944+ CCD detector. Data were then integrated and scaled using HKL3000 (Otwinowski and Minor, 1997). All Tpa1p structures and the structure of OGFOD1:Mn(II):NOG were determined by molecular replacement (MR) using the MR-PHASER (McCoy et al., 2007) subroutine in PHENIX (Adams et al., 2010) and the reported *S. cerevisiae* Tpa1p structure (PDB ID: 3KT4 [Kim et al., 2009]) as the search model. An OGFOD1:Mn(II):2,4-PDCA structure was subsequently determined by MR using the refined OGFOD1:Mn(II):NOG structure. Model building and refinement were performed iteratively using Coot (Emsley and Cowtan, 2004) and PHENIX until the decreasing R and R_{free} no longer converged. Mn(II), NOG, 2,4-PDCA, and IOX3 and water molecules were

Figure 8. Variations in the Active Site Metal Coordination Chemistry of Selected Human 2-Oxoglutarate Oxygenases

(A–G) Views from the active sites of (A) OGFOD1 (PDB ID: 4NHX), (B) termination and polyadenylation protein 1 (Tpa1p) (PDB ID: 4NHK, 4NHM), (C) HIF prolyl hydroxylase (PHD2) (PDB ID: 2G1M, 3HQR) (McDonough et al., 2006; Chowdhury et al., 2009), (D) a collagen-like prolyl 4-hydroxylase enzyme from *C. reinhardtii* (CrP4H) (PDB ID: 2JIG, 3GZE) (Koski et al., 2007, 2009), (E) *Pseudomonas aeruginosa* prolyl hydroxylase domain containing protein (PPHD) (PDB ID:4J25, 4IW3) (Scotti et al., 2014), (F) factor-inhibiting HIF (FIH) (PDB ID: 1H2N, 2Y0I) (Elkins et al., 2007; Yang et al., 2011), and (G) Human γ-butyrobetaine hydroxylase (BBOX) (PDB ID: 3N6W, 3O2G) (Tars et al., 2010). Waters/metals and residues/inhibitors/2OG are spheres and stick models, respectively. Distances are in angstroms. The metal ligating water (W1) is in red.

2,4-PDCA, pyridine-2,4-dicarboxylic acid; 4HG, *N*-[(4-hydroxy-8-iodoisoquinolin-3-yl)carbonyl]glycine; EF-Tu, elongation factor Tu; NOG, *N*-oxalylglycine.

modeled in the final stages of refinement based on the $F_{obs} - F_{calc}$ electron density maps.

ACCESSION NUMBERS

The PDB accession numbers for the structures of OGFOD1:NOG, OGFOD1: 2,4-PDCA, Tpa1p:NOG, Tpa1p:2,4-PDCA, and Tpa1p:IOX3 reported in this paper are 4NHX, 4NHY, 4NHL, 4NHK, and 4NHM, respectively.

SUPPLEMENTAL INFORMATION

Supplemental Information includes two figures, one table, and five 3D molecular models and can be found with this article online at http://dx.doi.org/10.1016/j.str.2015.01.014.

AUTHOR CONTRIBUTIONS

C.J.S. and M.A.M. directed the project; S.H. and J.S.S. prepared samples; S.H., J.S.S., and A.T. performed crystallography; S.H., J.S.S., and M.A.M. analyzed crystallographic data; S.H. and C.T. performed kinetics assays; Y.S.M.-T. and C.L. generated variants and performed yeast cell assays; W.G. prepared peptides for assays; W-S.A. provided technical advice; S.H., J.S.S., C.J.S., and M.A.M. wrote the manuscript.

ACKNOWLEDGMENTS

We thank the Japan Society for the Promotion of Science (S.H.), the Rhodes Trust (J.S.S.), the British Heart Foundation, the Wellcome Trust, and the Biotechnology and Biological Sciences Research Council for financial support; Ray Owens, Oxford Protein Production Facility, for contributing an OGFOD1 plasmid; the staff at Diamond Light Source; and P.J. Ratcliffe, M. Cockman, C.W. Pugh, and F.M. Ashcroft for discussions.

Received: November 19, 2014
Revised: January 13, 2015
Accepted: January 21, 2015
Published: February 26, 2015

REFERENCES

Adams, P.D., Afonine, P.V., Bunkóczi, G., Chen, V.B., Davis, I.W., Echols, N., Headd, J.J., Hung, L.W., Kapral, G.J., Grosse-Kunstleve, R.W., et al. (2010). PHENIX: a comprehensive Python-based system for macromolecular structure solution. Acta Crystallogr. D Biol. Crystallogr. *66*, 213–221.

Aik, W.S., McDonough, M.A., Thalhammer, A., Chowdhury, R., and Schofield, C.J. (2012). Role of the jelly-roll fold in substrate binding by 2-oxoglutarate oxygenases. Curr. Opin. Struct. Biol. *22*, 691–700.

Anand, R., Dorrestein, P.C., Kinsland, C., Begley, T.P., and Ealick, S.E. (2002). Structure of oxalate decarboxylase from *Bacillus subtilis* at 1.75 Å resolution. Biochemistry *41*, 7659–7669.

Ban, N., Beckmann, R., Cate, J.H., Dinman, J.D., Dragon, F., Ellis, S.R., Lafontaine, D.L., Lindahl, L., Lijas, A., Lipton, J.M., et al. (2014). A new system for naming ribosomal protein. Curr. Opin. Struct. Biol. *24*, 165–169.

Chowdhury, R., McDonough, M.A., Mecinovic, J., Loenarz, C., Flashman, E., Hewitson, K.S., Domene, C., and Schofield, C.J. (2009). Structural basis for binding of hypoxia-inducible factor to the oxygen-sensing prolyl hydroxylases. Structure *17*, 981–989.

Chowdhury, R., Candela-Lena, J.I., Chan, M.C., Greenald, D.J., Yeoh, K.K., Tian, Y.M., McDonough, M.A., Tumber, A., Rose, N.R., Conejo-Garcia, A., et al. (2013). Selective small molecule probes for the hypoxia inducible factor (HIF) prolyl hydroxylases. ACS Chem. Biol. *8*, 1488–1496.

Chowdhury, R., Sekirnik, R., Brissett, N.C., Krojer, T., Ho, C.H., Ng, S.S., Clifton, I.J., Ge, W., Kershaw, N.J., Fox, G.C., et al. (2014). Ribosomal oxygenases are structurally conserved from prokaryotes to humans. Nature *510*, 422–426.

Clifton, I.J., Hsueh, L.C., Baldwin, J.E., Harlos, K., and Schofield, C.J. (2001). Structure of proline 3-hydroxylase. Evolution of the family of 2-oxoglutarate dependent oxygenases. Eur. J. Biochem. *268*, 6625–6636.

Clifton, I.J., McDonough, M.A., Ehrismann, D., Kershaw, N.J., Granatino, N., and Schofield, C.J. (2006). Structural studies on 2-oxoglutarate oxygenases and related double-stranded β-helix fold proteins. J. Inorg. Biochem. *100*, 644–669.

Elkins, J.M., Hewitson, K.S., McNeill, L.A., Seibel, J.F., Schlemminger, I., Pugh, C.W., Ratcliffe, P.J., and Schofield, C.J. (2007). Structure of factor-inhibiting hypoxia-inducible factor (HIF) reveals mechanism of oxidative modification of HIF-1α. J. Biol. Chem. *278*, 1802–1806.

Emsley, P., and Cowtan, K. (2004). Coot: model-building tools for molecular graphics. Acta Crystallogr. D Biol. Crystallogr. *60*, 2126–2132.

Eriksson, M., Myllyharju, J., Tu, H., Hellman, M., and Kivirikko, K.I. (1999). Evidence for 4-hydroxyproline in viral proteins. Characterization of a viral prolyl 4-hydroxylase and its peptide substrates. J. Biol. Chem. *274*, 22131–22134.

Falnes, P.Ø., Klungland, A., and Alseth, I. (2007). Repair of methyl lesions in DNA and RNA by oxidative demethylation. Neuroscience *145*, 1222–1232.

Feng, T., Yamamoto, A., Wilkins, S.E., Sokolova, E., Yates, L.A., Münzel, M., Singh, P., Hopkinson, R.J., Fischer, R., Cockman, M.E., et al. (2013). Optimal translational termination requires C4 lysyl hydroxylation of eRF1. Mol. Cell *53*, 645–654.

Flagg, S.C., Giri, N., Pektas, S., Maroney, M.J., and Knapp, M.J. (2012). Inverse solvent isotope effects demonstrate slow aquo release from hypoxia inducible factor-prolyl hydroxylase (PHD2). Biochemistry *51*, 6654–6666.

Flashman, E., Bagg, E.A., Chowdhury, R., Mecinovic, J., Loenarz, C., McDonough, M.A., Hewitson, K.S., and Schofield, C.J. (2008). Kinetic rationale for selectivity toward N- and C-terminal oxygen-dependent degradation domain substrates mediated by a loop region of hypoxia-inducible factor prolyl hydroxylases. J. Biol. Chem. *283*, 3808–3815.

Flashman, E., Davies, S.L., Yeoh, K.K., and Schofield, C.J. (2010). Investigating the dependence of the hypoxia-inducible factor hydroxylases (factor inhibiting HIF and prolyl hydroxylase domain 2) on ascorbate and other reducing agents. Biochem. J. *427*, 135–142.

Ge, W., Wolf, A., Feng, T., Ho, C.H., Sekirnik, R., Zayer, A., Granatino, N., Cockman, M.E., Loenarz, C., Loik, N.D., et al. (2012). Oxygenase-catalyzed ribosome hydroxylation occurs in prokaryotes and humans. Nat. Chem. Biol. *8*, 860–962.

Gile, C., and Frömmel, C. (2001). STRAP: editor for STRuctural Alignments of Proteins. Bioinformatics *18*, 183–191.

Gorres, K.L., and Raines, R.T. (2010). Prolyl 4-hydroxylase. Crit. Rev. Biochem. Mol. Biol. *45*, 106–124.

Hausinger, R.P. (2004). FeII/alpha-ketoglutarate-dependent hydroxylases and related enzymes. Crit. Rev. Biochem. Mol. Biol. *39*, 21–68.

Henri, J., Rispal, D., Bayart, E., van Tilbeurgh, H., Séraphin, B., and Graille, M. (2010). Structural and functional insights into *Saccharomyces cerevisiae* Tpa1, a putative prolylhydroxylase influencing translation termination and transcription. J. Biol. Chem. *285*, 30767–30778.

Horton, J.R., Upardhyay, A.K., Qi, H.H., Zhang, X., Shi, Y., and Cheng, X. (2010). Enzymatic and structural insights for substrate specificity of a family of jumonji histone lysine demethylases. Nat. Struct. Mol. Biol. *17*, 38–43.

Jia, G., Fu, Y., and He, C. (2013). Reversible RNA adenosine methylation in biological regulation. Trends Genet. *29*, 108–115.

Kaelin, W.G., Jr., and Ratcliffe, P.J. (2008). Oxygen sensing by metazoans: the central role of the HIF hydroxylase pathway. Mol. Cell *30*, 393–402.

Katz, M.J., Acevedo, J.M., Loenarz, C., Galaqovsky, D., Liu-Yi, P., Pérez-Pepe, M., Thalhammer, A., Sekirnik, R., Ge, W., Melani, M., et al. (2014). Sudestada1, a *Drosophila* ribosomal prolyl-hydroxylase required for protein translation, cell homeostasis and organ growth. Proc. Natl. Acad. Sci. USA *111*, 4025–4030.

Keeling, K.M., Salas-Marco, J., Osherovich, L.Z., and Bedwell, D.M. (2006). Tpa1p is part of an mRNP complex that influences translation termination, mRNA deadenylation, and mRNA turnover in *Saccharomyces cerevisiae*. Mol. Cell. Biol. *26*, 5237–5248.

Kim, H.S., Kim, H.L., Kim, K.H., Kim, D.J., Lee, S.J., Yoon, J.Y., Yoon, H.J., Lee, H.Y., Park, S.B., Kim, S.J., et al. (2009). Crystal structure of Tpa1 from *Saccharomyces cerevisiae*, a component of the messenger ribonucleoprotein complex. Nucleic Acids Res. *38*, 2099–2110.

Klose, R.J., Kallin, E.M., and Zhang, Y. (2006). JmjC-domain-containing proteins and histone demethylation. Nat. Rev. Genet. *7*, 715–727.

Koivunen, P., Hirsilä, M., Günzler, V., Kivirikko, K.I., and Myllyharju, J. (2004). Catalytic properties of the asparaginyl hydroxylase (FIH) in the oxygen sensing pathway are distinct from those of its prolyl 4-hydroxylases. J. Biol. Chem. *279*, 9899–9904.

Koketsu, K., Shomura, Y., Moriwaki, K., Hayashi, M., Mitsuhashi, S., Hara, R., Kino, K., and Higuchi, Y. (2014). Refined regio- and stereoselective hydroxylation of L-pipecolic acid by protein engineering of L-proline cis-4-hydroxylase based on the X-ray crystal structure. ACS Synth. Biol. Published online August 29, 2014. http://dx.doi.org/10.1021/sb500247a.

Koski, M.K., Hieta, R., Böllner, C., Kivirikko, K.I., Myllyharju, J., and Wierenga, R.K. (2007). The active site of an algal prolyl 4-hydroxylase has a large structural plasticity. J. Biol. Chem. *282*, 37112–37123.

Koski, M.K., Hieta, R., Hirsilä, M., Rönkä, A., Myllyharju, J., and Wierenga, R.K. (2009). The crystal structure of an algal prolyl 4-hydroxylase complexed with a proline-rich peptide reveals a novel buried tripeptide binding motif. J. Biol. Chem. *284*, 25290–25301.

Landau, M., Mayrose, I., Rosenberg, Y., Glaser, F., Martz, E., Pupko, T., and Ben-Tai, N. (2005). Consurf 2005: the projection of evolutionary conservation scores of residues on protein structures. Nucleic Acids Res. *33*, W299–W302.

Larkin, M.A., Blackshields, G., Brown, N.P., Chenna, R., McGettigan, P.A., McWilliam, H., Valentin, F., Wallace, I.M., Wilm, A., Lopez, R., et al. (2007). Clustal W and Clustal X version 2.0. Bioinformatics *23*, 2947–2948.

Lee, C.Y., Stewart, E.V., Hughes, B.T., and Espenshade, P.J. (2009). Oxygen-dependent binding of Nro1 to the prolyl hydroxylase Ofd1 regulates SREBP degradation in yeast. EMBO J. *28*, 135–143.

Loenarz, C., and Schofield, C.J. (2008). Expanding chemical biology of 2-oxoglutarate oxygenases. Nat. Chem. Biol. *4*, 152–156.

Loenarz, C., and Schofield, C.J. (2011). Physiological and biochemical aspects of hydroxylations and demethylations catalyzed by human 2-oxoglutarate oxygenases. Trends Biochem. Sci. *36*, 7–18.

Loenarz, C., Sekirnik, R., Thalhammer, A., Ge, W., Spivakovsky, E., Mackeen, M.M., McDonough, M.A., Cockman, M.E., Kessler, B.M., Ratcliffe, P.J., et al. (2014). Hydroxylation of the eukaryotic ribosomal decoding centre affects translational accuracy. Proc. Natl. Acad. Sci. USA *111*, 4019–4024.

McCoy, A.J., Grosse-Kunstleve, R.W., Adams, P.D., Winn, M.D., Storoni, L.C., and Read, R.J. (2007). Phaser crystallographic software. J. Appl. Crystallogr. *40*, 658–674.

McDonough, M.A., Li, V., Flashman, E., Chowdhury, R., Mohr, C., Liénard, B.M., Zondlo, J., Oldham, N.J., Clifton, I.J., Lewis, J., et al. (2006). Cellular oxygen sensing: crystal structure of hypoxia-inducible factor prolyl hydroxylase (PHD2). Proc. Natl. Acad. Sci. USA *103*, 9814–9819.

Myllyharju, J., and Kivirikko, K.I. (2004). Collagens, modifying enzymes and their mutations in humans, flies and worms. Trends Genet. *20*, 33–43.

Namy, O., Duchateau-Nguyen, G., Hatin, I., Hermann-Le Denmat, S., Termier, M., and Rousset, J.P. (2003). Identification of stop codon readthrough genes in Saccharomyces cerevisiae. Nucleic Acids Res. *31*, 2289–2296.

Neidig, M.L., Brown, C.D., Light, K.M., Fujimori, D.G., Nolan, E.M., Price, J.C., Barr, E.W., Bollinger, J.M., Jr., Krebs, C., Walsh, C.T., et al. (2007). CD and MCD of CytC3 and taurine dioxygenase: role of the facial triad in α-KG-dependent oxygenases. J. Am. Chem. Soc. *129*, 14224–14231.

Otwinowski, Z., and Minor, W. (1997). Processing of X-ray diffraction data collected in oscillation mode. Methods Enzymol. *276*, 307–326.

Pang, H., Bartlam, M., Zeng, Q., Miyatake, H., Hisano, T., Miki, K., Wong, L.L., Gao, G.F., and Rao, Z. (2004). Crystal structure of human Pirin: an iron-binding nuclear protein and transcription cofactor. J. Biol. Chem. *279*, 1491–1498.

Pekkala, M., Hieta, R., Bergmann, U., Kivirikko, K.I., Wierenga, R.K., and Myllyharju, J. (2004). The peptide-substrate-binding domain of collagen prolyl 4-hydroxylases is a tetratricopeptide repeat domain with functional aromatic residues. J. Biol. Chem. *279*, 52255–52261.

Pollard, P.J., Loenarz, C., Mole, D.R., McDonough, M.A., Gleadle, J.M., Schofield, C.J., and Ratcliffe, P.J. (2008). Regulation of Jumonji-domain-containing histone demethylases by hypoxia-inducible factor (HIF)-1α. Biochem. J. *416*, 387–394.

Poppe, L., Tegley, C.M., Li, V., Lewis, J., Zondlo, J., Yang, E., Kurzeja, R.J., and Syed, R. (2009). Different modes of inhibitor binding to prolyl hydroxylase by combined use of X-ray crystallography and NMR spectroscopy of paramagnetic complexes. J. Am. Chem. Soc. *131*, 16654–16655.

Rispal, D., Henri, J., van Tilbeurgh, H., Graille, M., and Seraphin, B. (2011). Structural and functional analysis of Nro1/Ett1: a protein involved in translation termination in S. cerevisiae and in O_2-mediated gene control in S. pombe. RNA *17*, 1213–1224.

Rose, N.R., McDonough, M.A., King, O.N., Kawamura, A., and Schofield, C.J. (2011). Inhibition of 2-oxoglutarate dependent oxygenases. Chem. Soc. Rev. *40*, 4364–4397.

Saito, K., Adachi, N., Koyama, H., and Matsushita, M. (2010). OGFOD1, a member of the 2-oxoglutarate and iron dependent dioxygenase family, functions in ischemic signaling. FEBS J. *584*, 3340–3347.

Schofield, C.J., and Ratcliffe, P.J. (2004). Oxygen sensing by HIF hydroxylases. Nat. Rev. Mol. Cell Biol. *5*, 343–354.

Scotti, J.S., Leung, I.K., Ge, W., Bentley, M.A., Paps, J., Kramer, H.B., Lee, J., Aik, W.S., Choi, H., Paulsen, S.M., et al. (2014). Human oxygen sensing may have origins in prokaryotic elongation factor Tu prolyl-hydroxylation. Proc. Natl. Acad. Sci. USA *111*, 13331–13336.

Singleton, R.S., Liu-Yi, P., Formenti, F., Ge, W., Sekirnik, R., Fischer, R., Adam, J., Pollard, P.J., Wolf, A., Thalhammer, A., et al. (2014). OGFOD1 catalyzes prolyl hydroxylation of uS12 and is involved in translation control and stress granule formation. Proc. Natl. Acad. Sci. USA *111*, 4031–4036.

Steiner, R.A., Kalk, K.H., and Dijkstra, B.W. (2002). Anaerobic enzyme.substrate structures provide insight into the reaction mechanism of the copper-dependent quercetin 2,3-dioxygenase. Proc. Natl. Acad. Sci. USA *99*, 16625–16630.

Tars, K., Rumnieks, J., Zeltins, A., Kazaks, A., Kotelovica, S., Leonciks, A., Sharipo, J., Viksna, A., Kuka, J., Liepinsh, et al.. (2010). Crystal structure of human gamma-butyrobetaine hydroxylase. Biochem. Biophys. Res. Commun. *398*, 634–639.

Tian, Y.M., Yeoh, K.K., Lee, M.K., Eriksson, T., Kessler, B.M., Kramer, H.B., Edelmann, M.J., William, C., Pugh, C.W., Schofield, C.J., et al. (2011). Differential sensitivity of hypoxia inducible factor hydroxylation sites to hypoxia and hydroxylase inhibitors. J. Biol. Chem. *286*, 13041–13051.

Vranka, J.A., Sakai, L.Y., and Bächinger, H.P. (2004). Prolyl 3-hydroxylase 1, enzyme characterization and identification of a novel family of enzymes. J. Biol. Chem. *279*, 23615–23621.

Webby, C.J., Wolf, A., Gromak, N., Dreger, M., Kramer, H., Kessler, B., Nielsen, M.L., Schmitz, C., Butler, D.S., Yates, J.R., 3rd., et al. (2009). Jmjd6 catalyses lysyl-hydroxylation of U2AF65, a protein associated with RNA splicing. Science *325*, 90–93.

Wehner, K.A., Schütz, S., and Sarnow, P. (2010). OGFOD1, a novel modulator of eukaryotic translation initiation factor 2alpha phosphorylation and the cellular response to stress. Mol. Cell. Biol. *30*, 2006–2016.

Yang, M., Chowdhury, R., Ge, W., Hamed, R.B., McDonough, M.A., Claridge, T.D., Kessler, B.M., Cockman, M.E., Ratcliffe, P.J., and Schofield, C.J. (2011). Factor-inhibiting hypoxia-inducible factor (FIH) catalyzes the post-translational hydroxylation of histidinyl residues within ankyrin repeat domains. FEBS J. *278*, 1086–1097.

Yeh, T.L., Lee, C.S.Y., Amzel, L.M., Espenshade, P.J., and Bianchet, M.B. (2011). The hypoxic regulator of sterol synthesis Nro1 is a nuclear import adaptor. Structure *19*, 503–514.

Structure, Volume *23*

Supplemental Information

Structure of the Ribosomal Oxygenase OGFOD1 Provides Insights into the Regio- and Stereoselectivity of Prolyl Hydroxylases

Shoichiro Horita, John S. Scotti, Cyrille Thinnes, Yousef S. Mottaghi-Taromsari, Armin Thalhammer, Wei Ge, WeiShen Aik, Christoph Loenarz, Christopher J. Schofield, and Michael A. McDonough

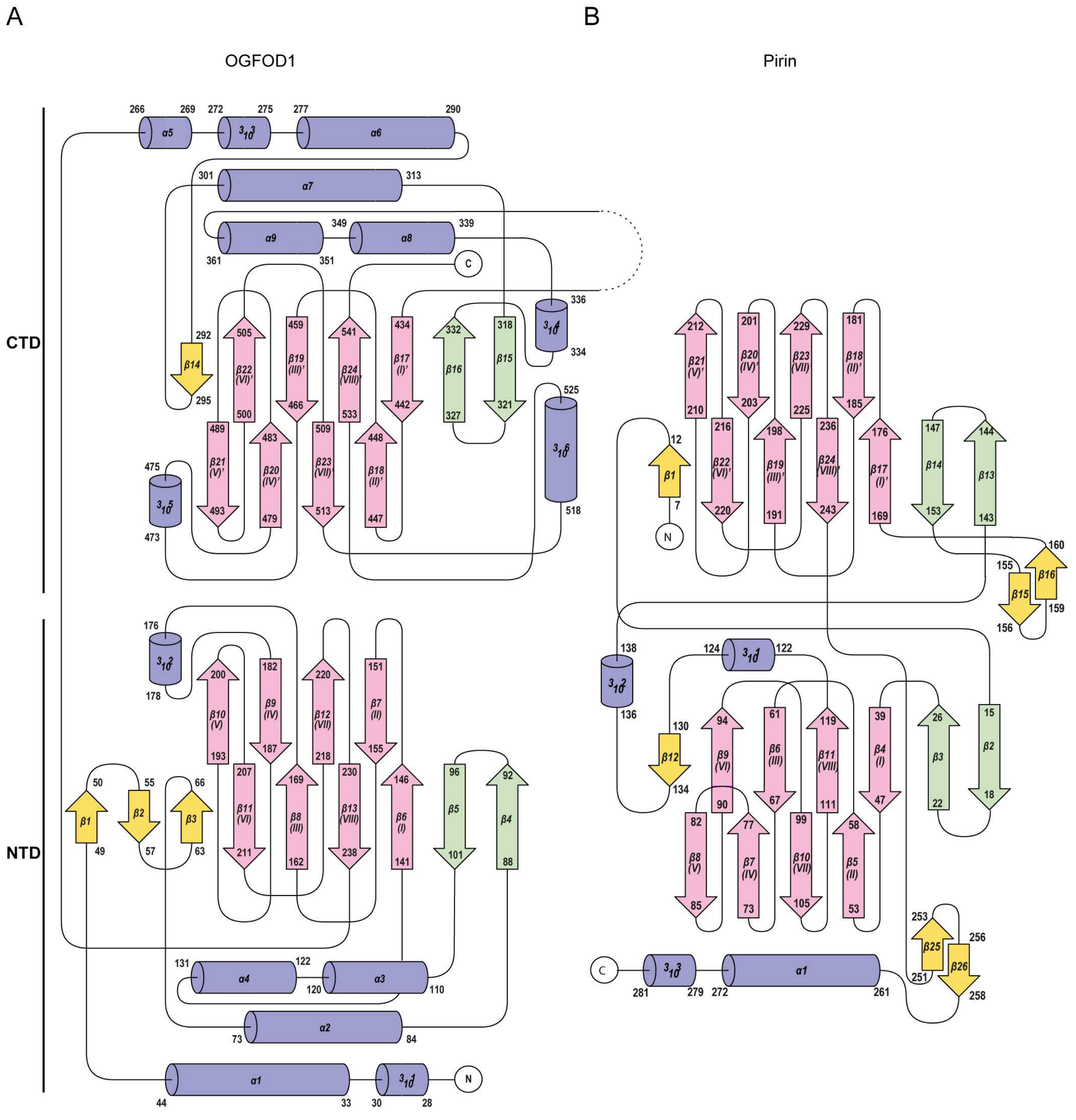

Figure S1, related to Figure 2. Topological fold diagrams of (A) OGFOD1 (PDB ID: 4NHX) and (B) Pirin (Pang et al., 2004) (PDB ID: 1J1L), α-helix and 3_{10}-helix (blue), β-strand (pink/green/yellow). The eight β-strands forming the core DSBH fold (pink), the β-strands forming the β4-β5 hairpin (green), all other strands (yellow) are shown. The eight DSBH β-strands of OGFOD1 are additionally labelled with Roman numerals as in Clifton *et al.* (Clifton et al., 2006). The long disordered 'acidic' loop is represented by a dashed line.

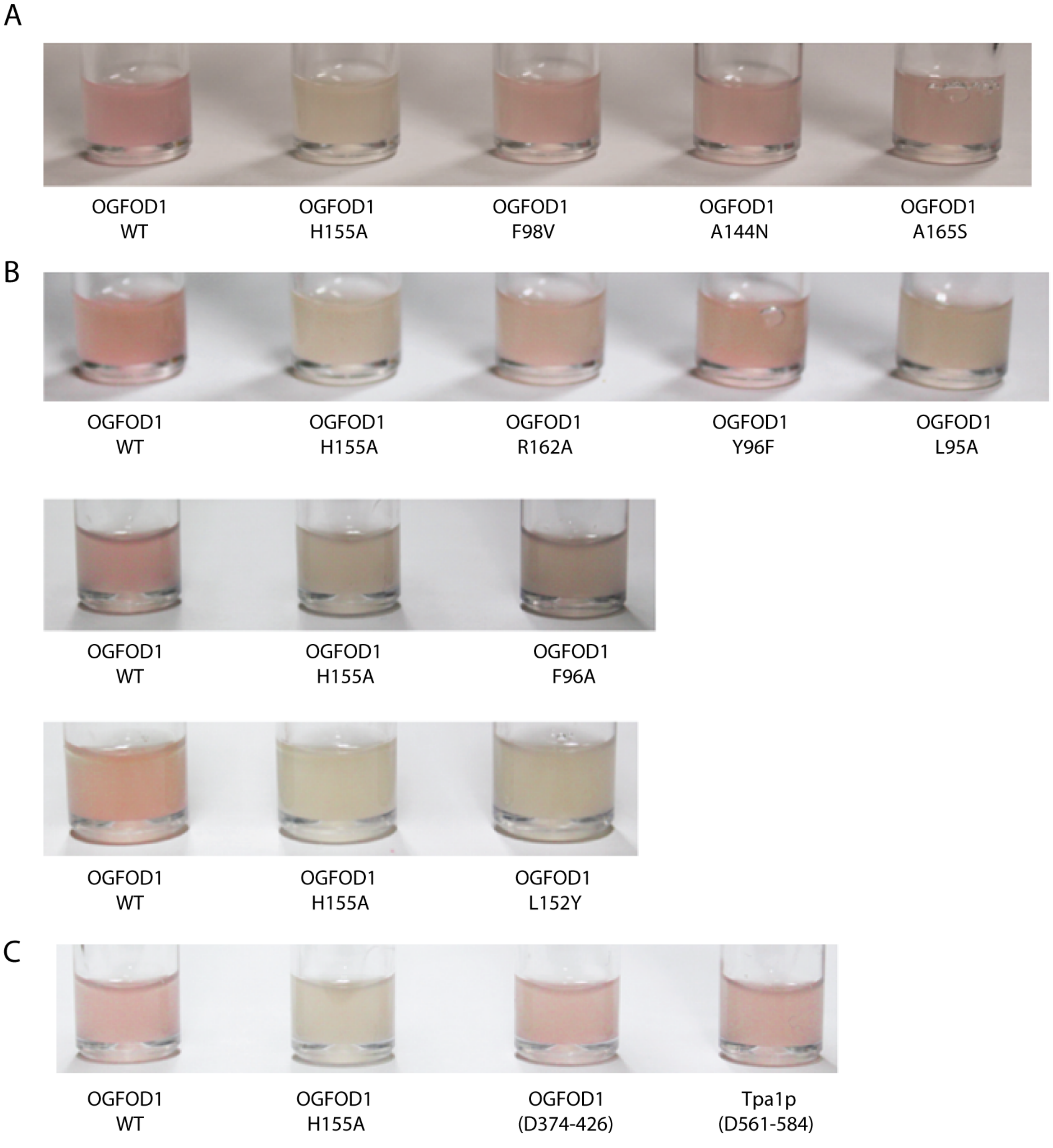

Figure S2, related to Figure 7. Yeast cell assays of OGFOD1 and Tpa1p variants for assessing yeast uS12 *trans*-3-hydroxylation. Di-hydroxylation of Pro64 was observed for Tpa1p activity but not OGFOD1 (Loenarz et al., 2014). We analysed the structures for active site differences between OGFOD1 and Tpa1p in pursuit of identifying residues responsible for mono- versus di-hydroxylation. Substitution of OGFOD1 residues to the corresponding Tpa1p residues (F98V, A144N, and A165S) (within 11 Å of the active site metal in the substrate binding groove) (Figure 7I) did not appear to affect *trans*-P3H activity in the cellular assays, suggesting these changes are tolerated (A) and that lack of di-hydroxylation activity by OGFOD1 may be influenced by factors other than/additional to immediate active site differences.

Table S1, related to Table 1. Details of crystallization conditions.

	OGFOD1:NOG[a]	OGFOD1:2,4-PDCA[a]	Tpa1p:NOG[a]	Tpa1p:2,4-PDCA[a]	Tpa1p:IOX3[a]
**Protein solution**	~10 mg/mL OGFOD1, 0.7 mM $MnCl_2$, 1.0 mM NOG, 1.0 mM C036 peptide	~10 mg/mL OGFOD1, 2.0 mM $MnCl_2$, 2.0 mM 2,4-PDCA	~9.5 mg/mL TPA1, 0.8 mM $MnCl_2$, 1.1 mM NOG	~6 mg/mL TPA1, 0.8 mM $MnCl_2$, 1.1 mM 2,4-PDCA	~6 mg/mL TPA1, 0.8 mM $MnCl_2$, 1.1 mM IOX3
**Reservoir solution**	0.1 M MIB buffer pH 6.5, 0.1 M glycine, 25% PEG 1500	0.1 M HEPES pH 7.0, 0.2 M $MgCl_2$, 20% PEG 6000	0.1 M succinic acid pH 7.0, 12% PEG 3350	0.1 M SPG buffer pH 8.0, 25% PEG 1500	0.2 M trisodium citrate, 20% PEG 3350

Abbreviations: PEG; polyethylene glycol, **SPG** buffer; succinic acid, phosphate, glycine, **MIB** buffer; malonic acid, imidazole, boric acid. C036 peptide; LEKLGIESKQPNSAIRKAVR($_D$-Cys)($_D$-Cys, $_D$-cysteine residue).
[a] All crystals were soaked in cryoprotectant containing reservoir solution diluted with 25% glycerol.

REFERENCES

Clifton, I.J., McDonough, M.A., Ehrismann, D., Kershaw, N.J., Granatino, N., Schofield, C.J. (2006). Structural studies on 2-oxoglutarate oxygenases and related double-stranded β-helix fold proteins. J. Inorg. Biochem. *100*, 644-669.

Loenarz, C., Sekirnik, R., Thalhammer, A., Ge, W., Spivakovsky, E., Mackeen, M.M., McDonough, M.A., Cockman, M.E., Kessler, B.M., Ratcliffe, P.J., et al. (2014). Hydroxylation of the eukaryotic ribosomal decoding centre affects translational accuracy. Proc. Natl. Acad. Sci. USA *111*, 4019-4024.

Pang, H., Bartlam, M., Zeng, Q., Miyatake, H., Hisano, T., Miki, K., Wong, L.L. Gao, G.F., Rao, Z. (2004). Crystal structure of human Pirin: an iron-binding nuclear protein and transcription cofactor. J. Biol. Chem. *279*, 1491-1498.
